# Connexin 43 gap junction-mediated astrocytic network reconstruction attenuates isoflurane-induced cognitive dysfunction in mice

**DOI:** 10.1186/s12974-022-02424-y

**Published:** 2022-03-07

**Authors:** Rui Dong, Yuqiang Han, Linhao Jiang, Shuai Liu, Fujun Zhang, Liangyu Peng, Zimo Wang, Zhengliang Ma, Tianjiao Xia, Xiaoping Gu

**Affiliations:** 1 Department of Anesthesiology, Affiliated Drum Tower Hospital, Medical School of Nanjing University, No. 321 Zhongshan Road, Nanjing, China; 2 Medical School, Nanjing University, No. 22 Hankou Road, Nanjing, China; 3 Jiangsu Key Laboratory of Molecular Medicine, Nanjing University, Nanjing, China

**Keywords:** Isoflurane, Cognitive impairment, Connexin 43, Gap junctions, Astrocytic network, Neuroinflammation

## Abstract

**Background:**

Postoperative cognitive dysfunction (POCD) is a common complication following anesthesia and surgery. General anesthetic isoflurane has potential neurotoxicity and induces cognitive impairments, but the exact mechanism remains unclear. Astrocytes form interconnected networks in the adult brain through gap junctions (GJs), which primarily comprise connexin 43 (Cx43), and play important roles in brain homeostasis and functions such as memory. However, the role of the GJ-Cx43-mediated astrocytic network in isoflurane-induced cognitive dysfunction has not been defined.

**Methods:**

4-month-old male C57BL/6 mice were exposure to long-term isoflurane to induce cognitive impairment. To simulate an in vitro isoflurane-induced cognitive dysfunction‐like condition, primary mouse astrocytes were subjected to long-term isoflurane exposure. Cognitive function was assessed by Y-maze and fear conditioning tests. Western blot was used to determine the expression levels of different functional configurations of Cx43. The morphology of the GJs-Cx43 was evaluated by immunofluorescence staining. Levels of IL-1β and IL-6 were examined by ELISA. The ability of GJs-Cx43-mediated intercellular communication was examined by lucifer yellow dye transfer assay. Ethidium bromide uptake assays were used to measure the activity of Cx43 hemichannels. The ultrastructural morphology of astrocyte gap junctions and tripartite synapse were observed by transmission electron microscopy.

**Results:**

After long-term isoflurane anesthesia, the GJs formed by Cx43 in the mouse hippocampus and primary mouse astrocytes were significantly reduced, GJs function was impaired, hemichannel activity was enhanced, the levels of IL-1β and IL-6 were increased, and mice showed significant cognitive impairment. After treatment with the novel GJ-Cx43 enhancer ZP1609, GJ-Cx43-mediated astrocytic network function was enhanced, neuroinflammation was alleviated, and ameliorated cognition dysfunction induced by long-term isoflurane exposure. However, ZP1609 enhances the astrocytic network by promoting Cx43 to form GJs without affecting hemichannel activity. Additionally, our data showed that long-term isoflurane exposure does not alter the structure of tripartite synapse.

**Conclusion:**

Our results reveal a novel mechanism of the GJ-Cx43-mediated astrocytic network involved in isoflurane-induced neuroinflammation and cognitive impairments, which provides new mechanistic insight into the pathogenesis of POCD and identifies potential targets for its treatment.

**Supplementary Information:**

The online version contains supplementary material available at 10.1186/s12974-022-02424-y.

## Background

General anesthetics are indispensable for surgical procedures in preclinical and clinical work. Ideally, anesthetics work by causing temporary and reversible loss of consciousness and reactivity without additional side effects. General anesthetics, however, alter typical awake brain activity by acting on multiple mechanisms. Furthermore, the effects of anesthetics can last beyond the duration of anesthesia and lead to behavioral and cognitive manifestations [1, 2]. Recent advances in basic and clinical neuroscience suggest that anesthesia exposure increases the risk of developing Alzheimer’s disease and other forms of dementia [3]. Isoflurane, one of the most widely used anesthetics in preclinical and clinical settings, has both acute and chronic effects on brain function [4, 5]. Previous studies have confirmed that mice have learning and memory impairment after long-term isoflurane exposure (5–6 h) [6–9], which indicates that long-term isoflurane anesthesia contributes to postoperative cognitive dysfunction (POCD). POCD is a recognized clinical phenomenon of cognitive deficiency after anesthesia and surgery due to various factors, especially in the geriatric surgical population [10]. The morbid state not only results in a decreased quality of life but also facilitates the exacerbation of the disease. Neuroinflammation, which is principally driven by microglia and astrocytes, is a predominant neuropathological hallmark of POCD [11]. Astroglia amplify microglia-mediated neuroinflammation and result in the feedback loop of neuroinflammatory reactions [12]; furthermore, previous studies have confirmed that astrocytes are critically involved in the initiation and development of POCD [13–15].

Astrocytes are the most populous glial cell type in the central nervous system (CNS) and comprise nearly half the volume of the adult mammalian brain, dynamically interact with neurons and impact their activity and survival; thus, they are critical for regulating the brain microenvironment. The potential neuroprotective influence of astrocytes on neurons is broad because astrocytes are densely interconnected by gap junctions (GJs) that are composed primarily of the protein connexin 43 (Cx43) and can function as a broader network of cells rather than being isolated as single-cell units [16]. Astrocytic networks play an important role in brain homeostasis and repair following injury [16, 17]. An intact astrocytic network allows astrocytes to function more efficiently. Disruption of the network could serve to amplify even small pathological insults. For example, mice without astrocytic coupling show increased susceptibility to the experimental generation of epileptiform events [18]. In mouse models of ischemic stroke, mice lacking astrocytic Cx43 have a larger volume of tissue damage [19]. Disruption of the astrocyte network can impair energy metabolism in neurons and impair long-term plasticity [20]. However, pharmacologic enhancement of Cx43 gap junctional coupling between astrocytes is protective in stroke mouse models, decreasing the overall volume of the infarcted region [21]. The above-mentioned findings establish a gap junction-mediated astrocytic network as a promising target for neuroprotective therapies, controlling the maintenance of cerebral homeostasis.

Recently, there has been some evidence that GJs-Cx43-mediated astrocytic networks are involved in the progression of neurodegenerative disease [16, 22, 23]. However, the biological function of GJs-Cx43-mediated astrocytic networks during the pathophysiology of POCD is still unclear. Therefore, in this study, we aimed to elucidate the expression pattern and biological functions of the GJs-Cx43-mediated astrocytic network in isoflurane-induced cognitive dysfunction by using a variety of in vivo and cell-based approaches and to investigate whether targeting GJs-Cx43 can improve cognition in mice exposed to prolonged isoflurane anesthesia.

## Materials and methods

### Animals

Four-month-old male C57BL/6 mice (Weitong Lihua Experimental Animal Technology Co., Ltd., China) were housed in a standard condition under a 12-h light/12-h dark cycle (lights on at 8 a.m.) at constant temperature (22 + 1 °C) and relative humidity (50 ± 10%). Animals were housed in groups and allowed free access to standard mouse chow and water ad libitum. The experiments began after all the animals acclimated to the experimental environment for 2 weeks. All animal procedures were conducted following the Guidelines for Care and Use of Laboratory Animals of Nanjing University and all efforts were made to minimize animal suffering. The study protocol was approved by the Ethics Committee of the Affiliated Drum Tower Hospital, Medical School of Nanjing University (license No. 2020AE01110).

### Isoflurane general anesthesia

**Fig. 1:**
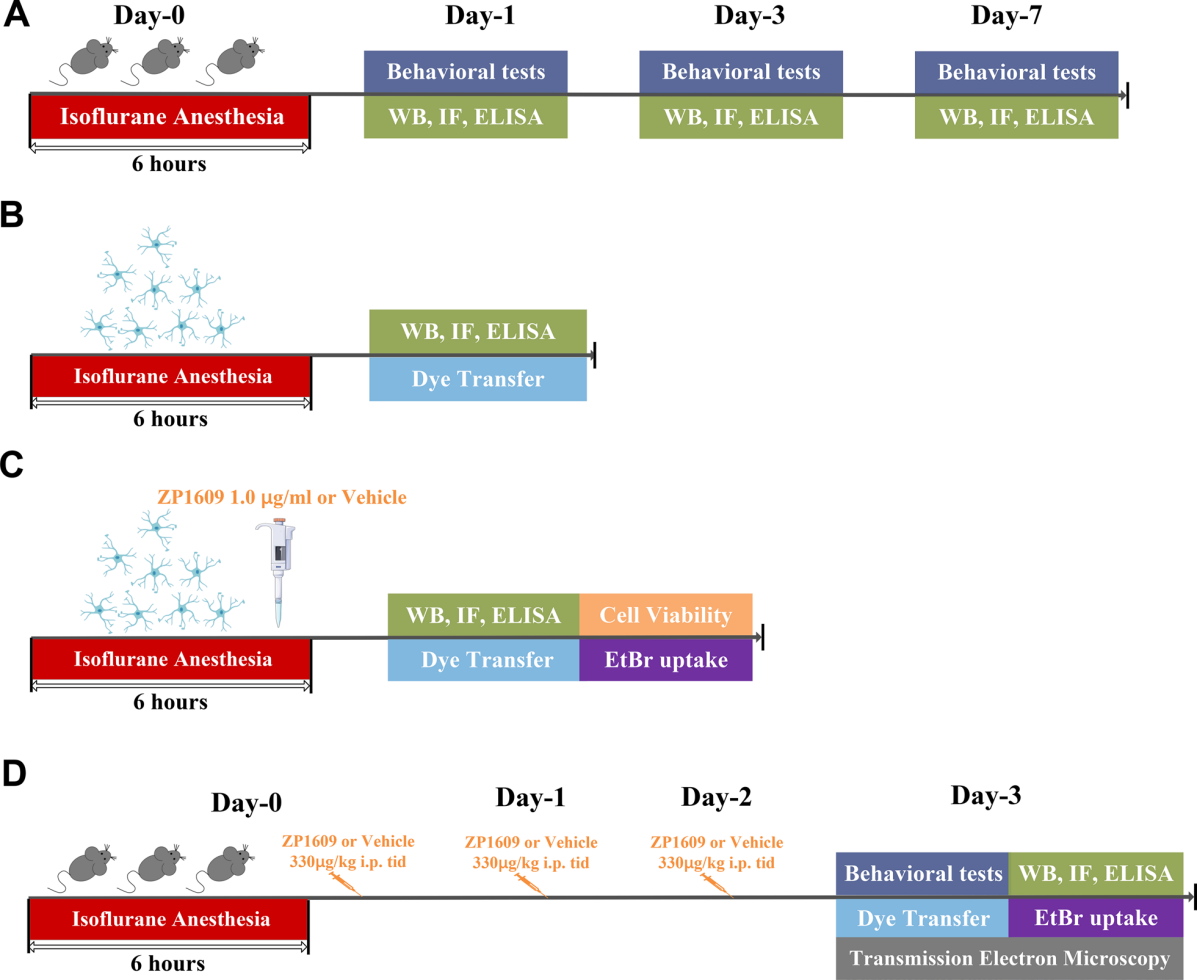
Graphic illustration of the experimental procedure. **a** In this section, the mice underwent behavioral tests on days 1, 3, and 7 after long-term isoflurane exposure. Hippocampus was immediately harvested at the end of the behavioral testing, and ELISA analysis for IL-1β and IL-6 levels was performed. The changes of Cx43 configuration were measured by immunofluorescence and Western blot. **b** The primary astrocytes were harvested 6 h after isoflurane exposure, and immunofluorescence and Western blot were performed to detect alterations of the Cx43 configuration. ELISA was performed to measure the level of IL-1β and IL-6 expression. Scrape-loading dye transfer was used to evaluate the biological function of Cx43 gap junction channels. **c** Schematic diagram of in vitro Cx43 gap junctions rescue experiment. Improvement of astrocyte pathological changes induced by long-term isoflurane anesthesia was observed by administration of ZP1609, a specific enhancer of the Cx43 gap junction. **d** Schematic diagram of in vivo Cx43 gap junctions functional rescued experiments. For GJs-Cx43-mediated astrocytic network intervention, mice were treated with ZP1609 (dosage: 300 µg/kg every time, three times/day) by intraperitoneal injection for 3 consecutive days after the mice model was established. Behavioral tests were conducted on day 3 after anesthesia. ELISA was performed to confirm the expression levels of IL-1β and IL-6. The changes of Cx43 configuration were measured by immunofluorescence and Western blot. Dye transfer was used to evaluate the biological function of Cx43 gap junction channels. Ethidium bromide (EtBr) uptake assays were used to measure the activity of Cx43 hemichannels. Transmission electron microscopy was used to observe ultrastructural alterations of astrocyte gap junction in mice hippocampal tissue

Animal anesthesia was performed using an inhalant gas mixture of isoflurane and air in reference to our previous study [6]. Mice were placed in a chamber with 4.2% isoflurane (license No. H20020267, Lunan Better Pharmaceutical Co., Ltd., China) for induction and 1.5% isoflurane in 100% oxygen at a flow rate of 2–3 l/min for maintenance for 6 h (from 9:00 pm to 3:00 am in the dark cycle). During isoflurane exposure, an anesthesia monitor was used to continuously monitor the concentration of isoflurane in the chamber, and respiration was observed to prevent respiratory depression. Mice were placed on a heating pad support to keep body temperature within 36.5 ± 0.5 °C, monitored with a rectal temperature probe. The same procedure was performed for the control animals, but not exposed to the isoflurane. Efforts were made to reduce the number of animals used and to minimize animal suffering. For GJs-Cx43-mediated astrocytic network intervention, mice were treated with ZP1609 (a specific enhancer of gap junctions, also known as danegaptide, GLPBIO, Cat: GC16618) by intraperitoneal injection for 3 consecutive days after the long-term isoflurane anesthesia. The schematic diagram of the animals’ experimental procedure is summarized in Fig. 1a and d.

Astrocyte anesthesia was carried out at 37 °C and in a 5% CO_2_ incubator. In brief, the incubator was pre-filled with 5% isoflurane mixed with high-flow mixed gases (5% CO_2_, 5 l/min), isoflurane concentration was subsequently diminished to 1.5% to maintain anesthesia for 6 h. Control astrocytes were treated in the same manner but without isoflurane. The flow of the in vitro experimental procedure is illustrated in Fig. 1b and c.

### Behavioral assessments

Behavioral assessments (12–18 mice per group) including fear conditioning test (FCT) and Y-Maze test were performed. All behavioral assessments were performed between 9:00 pm and 3:00 am in the dark cycle.

For FCT, in the training phase, each mice was left undisturbed for 3 min in the training box (21 cm long, 21 cm diameter, 26 cm elevated white plastic chamber), followed by two pairs of tone and electrical foot-shock stimuli. A loop of sound and electrical foot-shock stimuli consisted of a 30 s tone (75 dB, 1000HZ) and an inescapable 0.8 mA electrical foot-shock in the final 2 s. The pairs of tone and electrical foot shock stimuli were separated by intervals of 60 s. Sixty seconds after the last foot-shock, the mice were returned to their home cages. Contextual and tone fear memory was evaluated 24 h after training. In the contextual test, mice were placed in the training box and exposed to the context for 5 min without any stimulus. Mice that have learned the association between the training environment and the electrical shock will exhibit increased freezing. In the tone test, which was performed 2 h after the contextual test, mice were placed in the test box (21 × 21 × 26 cm black and white plastic chamber) to adapt for 3 min followed a 30 s tone (75 dB, 1000 HZ). One minute after the end of the tone stimulus, the experiment was terminated. After each test, 75% alcohol was used to clean the chamber to eliminate olfactory cues. Freezing was defined as lack of any visible movement except respiration, and it was monitored by the Tracking Master V3.0.

Spatial working memory was assessed by recording spontaneous alternation in the Y‐maze test. Mice with good working spatial memory were expected to enter a new arm of the maze without immediate reentry to a previously visited arm. Y-maze consists of three long, wide, and high arms of 35, 10, and 15 cm, which are the A arm, B arm, and C arm, respectively. Each mouse was placed in the center of the symmetrical Y-maze and allowed to explore the three arms freely for 8 min. EthoVision XT 11.5 was used to record the sequence and the total number of arms entered. An arm entry was defined as the entry of four limbs and tail into one arm of the Y-maze [24]. Unrepeated consecutive arm entries (i.e., ABC, BCA, CAB) were defined as success triad combination. The percentage of spontaneous alteration was calculated as (number of success triad combination)/(total number of arm entries − 2) × 100.

### Primary astrocyte culture

In this study, we want to clear the role of GJs-Cx43 on the change of astrocytic network caused by long-term isoflurane exposure. To avoid the influence caused by other cells or other situations on the expression of Cx43, the purified primary cultured astrocytes are used as the research object in this study, for they are the predominant cells expressed Cx43 in brain [25, 26]. Primary astrocytes were prepared from neonatal mice (within 48 h) were harvested under sterile conditions, and the protocol used was described previously [27]. Briefly, the cortical hemispheres were cut into pieces after careful removal of the meninges and then digested with trypsin (0.25%) for 10 min, filtered by centrifugation, and resuspended in DMEM medium (KeyGEN BioTech, China, Cat: KGM12800-500) containing 10% fetal bovine serum (Biological Industries, Cat: 04-001-1ACS). Cells were maintained at 37 °C with 5% CO_2_ in a humidified incubator. Fresh medium (DMEM supplemented with 10% fetal bovine serum) was changed twice a week. Primary astrocytes reached subconfluence at 7–8 days in vitro. When cultured to 95% confluence, astrocytes were purified by shaking the culture flask vigorously to remove other cells such as oligodendrocytes and microglia. GFAP immunofluorescence staining was used to identify primary astrocytes purity (Additional file 1: Fig. S1). Experiments were repeated with a different culture established from different breeding pairs.

### Immunofluorescence staining

Brain sections (16 μm) were prepared using a Leica CM3050 S Research cryostat (Leica Microsystems, Wetzlar, Germany) and stored at − 20 °C until immunofluorescence staining. In brief, the sections were permeabilized using 0.1% Triton X-100 for 30 min, followed by blocking with 10% goat serum for 60 min at room temperature. Incubation with rabbit polyclonal Cx43 (1:400, Sigma, Cat: C6219) was accomplished overnight at 4 °C. Alexa Fluor^®^ 594-conjugated goat anti-rabbit secondary antibodies (1:1000, Abcam, Cat: ab150080) was then added and the sections were incubated at room temperature for 90 min. Nuclei were stained with 4ʹ,6-diamidino-2-phenylindole dihydrochloride (DAPI) (Beyotime, Cat: P0131). A minimum of three randomly selected areas from each section of each sample was analyzed by the Thunder Imaging System (Leica, Germany). The polymorph assignment of Cx43 was verified according to reference, which GIs-Cx43 was defined as thresholded fluorescence puncta with an area ≥ 1 μm^2^ [27]. Percent fluorescence associated with GIs-Cx43 puncta was determined by dividing the area associated with GIs-Cx43 puncta by total fluorescence area.

For the immunofluorescence staining of astrocyte cultures, the treated cells on slides were fixed with 4% paraformaldehyde for 15 min after washing with PBS. Cell membranes were permeabilized by incubation with 0.1% Triton X-100 in PBS for 15 min at room temperature. Non-specific binding was blocked with 10% goat serum for 60 min. Primary antibodies were incubated at 4 °C overnight, and secondary antibodies were incubated for 90 min at room temperature, followed by DAPI staining. The antibody information is described in the preceding paragraph. Fluorescence was analyzed by using ImageJ software version 1.53c (National Institutes of Health, Bethesda, MD, USA). This experiment was conducted with a six-time repetition using different cultures established from different breeding pairs. A minimum of three randomly selected fields were analyzed for each group from independent experiments.

### Dye transfer in vivo

To determine whether gap junctions in the hippocampus are functional, we analyzed the diffusion of gap junctions permeable dye lucifer yellow, infused as described above [28]. Briefly, mice were anesthetized with 1% sodium pentobarbital before being placed in the stereotactic surgery frame (Ruiwode Life Technology Co., Ltd., Shenzhen, China). Intrahippocampal injection of lucifer yellow (Sigma-Aldrich, Cat: L0259) was performed at the following site: bregma − 2.5 mm, lateral 2 mm from the midline, 2.0 mm ventral to the dura [29]. After needle placement, 2 μl of a solution containing lucifer yellow (5 mg/ml) was infused bilaterally at a continuous rate of 0.25 μl/min. At 30 min after the infusion, the animals were perfused and brains sectioned (50 μm) on a vibratome (VT 1200S; Leica) as previously reported [30]. Intercellular diffusion of fluorescent molecules was captured with the Thunder Imaging System (Leica, Germany) and the distance of diffusion was analyzed with Image J 1.53c. Analysis was performed on three images of randomly selected areas from six different mice per group.

### Scrape-loading dye transfer in vitro

Gap junction-mediated intercellular coupling was evaluated by transfer of lucifer yellow dye in a scrape-loading assay as described previously with minor modifications [31]. Briefly, astrocytes were seeded on 12-well culture plates and incubated at 37 °C and 5% humidified atmosphere. Only cells at 95–100% confluence were used for the experiments herein. Astrocytes were subjected to isoflurane exposure for 6 h. Afterward, ZP1609 1 μg/ml in PBS was added to the culture medium at a final concentration of 1 µg/ml and then incubated at 37 °C for 30 min. Subsequently, the plates were rapidly and gently washed with PBS three times. The astrocyte monolayers were scratched with a sterile 5 ml syringe tip to form a cell-free area approximately 0.5 mm wide in order to allow the dye to enter the cells. Next, 1 ml 0.4% Lucifer yellow was added and incubated at 37 °C for an additional 30 min in the growth media to allow the loaded dye to transfer to adjoining cells. Excess dye was washed off with two rinses of FBS-free DMEM. Gap junction-coupled cells were imaged using the Thunder Imaging System (Leica, Germany) and the distance of Lucifer yellow diffusion was measured by the Image J 1.53c. Three random images were recorded for each plate and means were considered. Six independent experiments were performed. Another set of astrocytes without a scratch wound was stained simultaneously, to elicit background fluorescence.

### Dye uptake assay

Dye uptake assay using ethidium bromide (EtBr) was done to observe the activity of Cx43 hemichannels according to reference [32]. Briefly, for the in vitro experiments, astrocytes were inoculated in 24-well plates and subsequent manipulations were performed when cells grown to 70% confluence. The cells were washed twice with Locke’s solution and incubated with 0.01% EtBr in Locke’s solution for 10 min at 37 °C. The cells were washed again with Locke’s solution and fixed with 4% paraformaldehyde. Subsequently, the cells were proceeded with normal immunofluorescence protocol as described above to probe with anti-GFAP antibody (1:100, Cell Signaling Tech, Cat: 3670S). Nuclei were stained with DAPI. To evaluate the long-term isoflurane exposure and ZP1609 effects on the cellular hemichannel activity of a more integrated system, we investigated the EtBr uptake in astrocytes in acute hippocampal slices. Coronal brain sections (20 µm) were made using a vibratome (VT 1200S; Leica) filled with 4 °C artificial CSF. Acute slices were incubated with a solution containing 0.01% EtBr (Sigma-Aldrich, Cat: E7637) for 5 min, followed by washing with artificial CSF three times for 5 min each. The sections were then fixed with 4% paraformaldehyde for 30 min, GFAP and DAPI staining was performed as described above. Dye uptake level was calculated as the subtraction (F − F0) between the fluorescence (F) from the nuclei of GFAP-positive cell and the background fluorescence (F0) were detected. At least three fields were selected in every slice. Cells and brain sections were imaged using Thunder Imaging System (Leica, Germany) and the intensity of the EtBr was quantified using Image J 1.53c. Six fields were analyzed for each group from six independent experiments.

### Preparation of total protein lysates and 1% Triton X-100-soluble and insoluble fractions from the hippocampus

The Cx43 conformation that forms the gap junctions possesses the ability to be insoluble in 1% Triton X-100 [33, 34]. Based on this rationale, the conformations of different functional states of Cx43 were extracted and isolated according to reference [35]. For the preparation of total protein lysates, hippocampus tissues or astrocytes were collected for protein extraction by RIPA lysis buffer (P0013B, Beyotime) containing proteinase and phosphatase inhibitors (EMD Millipore, Cat: 524625). The samples were centrifuged at 12,000 rpm for 15 min after 30 min of lysis on ice. Total protein fractions were quantified using the BCA kit (Thermo Fisher, Cat: 34580). For the preparation of 1% Triton X-100-insoluble and soluble fractions, equal weight hippocampus tissues were homogenized in equal volumes of 1% Triton X-100 in immunoprecipitation buffer containing proteinase and phosphatase inhibitors. The samples were centrifuged at 12,000 rpm for 30 min at 4 °C after 30 min of lysis on ice. The supernatant was collected as the soluble fraction (non-GJs-Cx43). Precipitated protein was resuspended in an equal volume of 1% Triton X-100 in immunoprecipitation buffer containing 4 M urea. After sonication, the mixture was incubated at room temperature for 30 min. Subsequently, samples were centrifuged at 12,000 rpm at 4 °C for 30 min and the supernatant (GJs-Cx43) was collected. Total protein fractions were quantified using the BCA kit (Beyotime, Cat: P0009).

### Western blotting

Each lane was loaded with the same amount of protein (20 μg for tissue protein and 15 μg for cellular protein) after detecting the protein concentration, which was separated on an SDS-PAGE gel, and transferred onto a nitrocellulose membrane. The blots were incubated overnight at 4 °C with primary antibody rabbit polyclonal Cx43 (1:16,000, Sigma, Cat: C6219) and rabbit monoclonal antibody β-tubulin (1:7500, Beyotime, Cat: AF1216) for 2 h. Immunoreactive proteins were visualized by chemiluminescent solution (Thermo Fisher, Cat: 34580). β-Tubulin was used as the internal reference gene for normalization. The band intensities were measured to perform densitometric analysis using ImageJ software version 1.53c (National Institutes of Health, Bethesda, MD, USA).

### Perfusion and tissue preparation for electron microscopy

Electron microscopy experiments were conducted regarding the literature [36]. Briefly, after perfusion, the hippocampal tissue was cut into small blocks (∼ 1 mm^3^) and post-fixed overnight in the fixation solution and stored in 1:10 dilution of the same fixative in 0.1 M phosphate buffer. Randomly selected ultrathin sections were stained with 2% uranyl acetate and 2.6% lead citrate and examined using a transmission electron microscope (HT7800, HITACHI, Tokyo, Japan). Astrocyte gap junctions structure analysis was carried out according to previous literature minor modifications [37]. Two adjacent astrocytes were first located and pictures of the two close membranes were taken. Then, the integrity of the structure was evaluated and the width of gap was randomly measured at ten points with ImageJ software. Four pairs of astrocytes in each mouse were analyzed. Investigators were blinded regarding animal genotypes during processing and analysis. The electron microscopy image processing and analysis were performed by a pathologist blind to the group assignment of the animals.

### Cell viability assays

Cell viability was analyzed using a CCK-8 cell counting kit (Vazyme Biotech Co., Ltd, Cat: A311-02-AA) according to the manufacturer’s instructions. Primary astrocytes seeded in the 96-well plate were treated with ZP1609 at different concentrations (0 to 10 µg/ml) for 30 min, and then 10 μl CCK-8 reagent was added to each pore. After incubation in a cell incubator for 2 h, the absorbance at 450 nm was detected by a Multiskan GO Microplate Spectrophotometer (Thermo Scientific, USA). Cell viability was represented as a percentage of the control.

### Inflammatory factors content assays

Neuroinflammation was assessed by measuring the inflammatory cytokines in the hippocampus and astrocytes. Interleukin-1β (IL-1β) and interleukin-6 (IL-6) were measured using a commercially available ELISA kit (IL-1β: Invitrogen, Cat: 88-7013; IL-6: Invitrogen, Cat: 88-7064), according to the manufacturer's instructions. For the assay, 100 μl of culture supernatants were added to an ELISA plate well. Experiment results were repeated for at least three independent measurements.

### Statistical analysis

All data are presented as the mean ± SEM. Student’s *t*-test and analysis of variance (ANOVA) with post hoc tests were used for statistical comparison when appropriate. SPSS 22.0 (IBM, Armonk, USA) and GraphPad Prism software 8.0.1 (GraphPad Software, CA, USA) were applied to analyze the data and graphing, respectively. A value of *p* < 0.05 was deemed statistically significant.

## Results

### Long-term isoflurane anesthesia induces cognitive decline and induces inflammatory responses in hippocampus and astrocytes

**Fig. 2:**
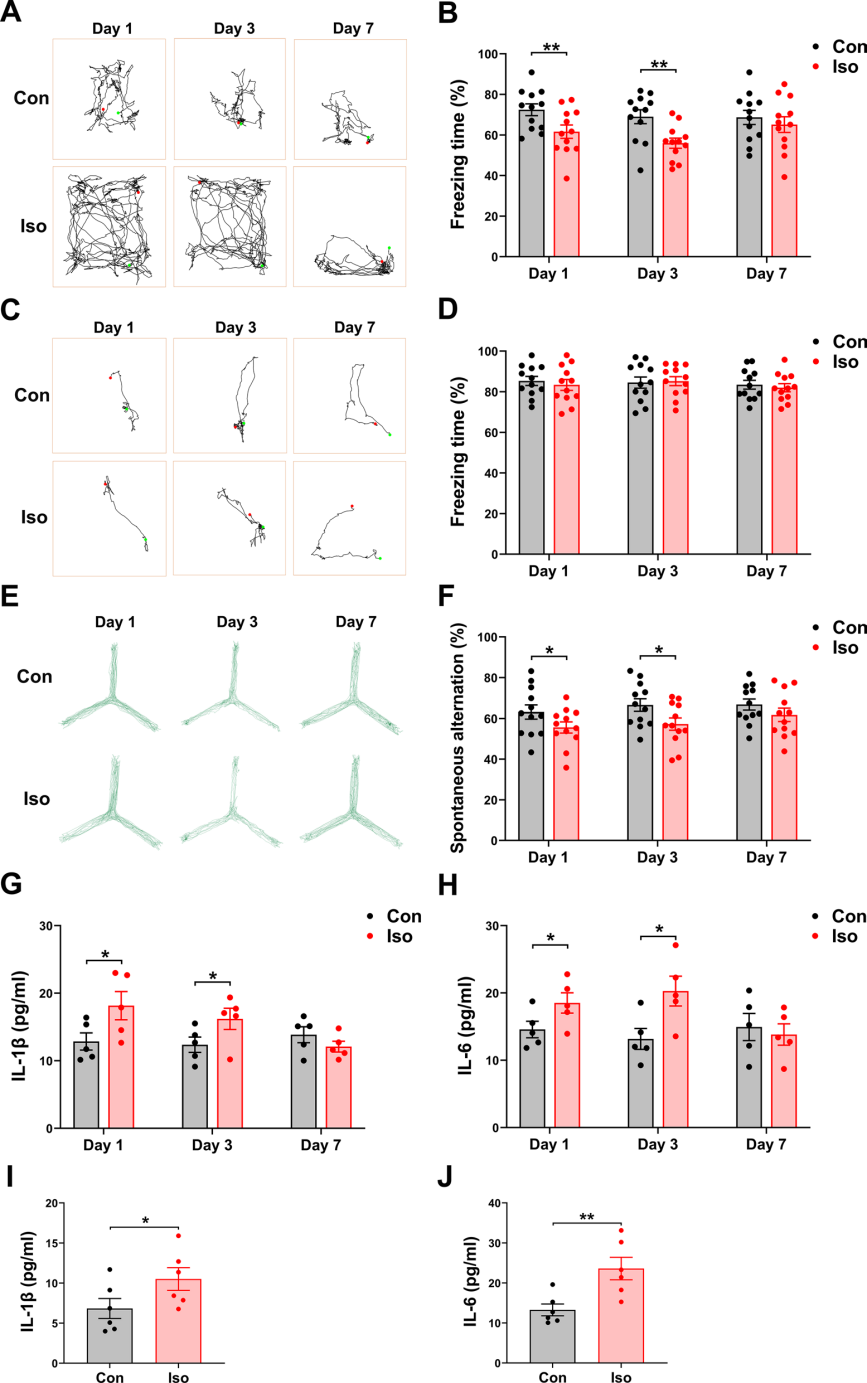
Long-term isoflurane anesthesia induces central neuroinflammation and cognitive dysfunction in mice. **a** Representative trajectories of each group in the context test of FCT. **b** At day 1 and 3 after long-term isoflurane anesthesia, in the context test of FCT, the percentage of freezing time was significantly reduced in the mice of isoflurane (Iso) group compared with control (Con) group. **c** Representative trajectories of each group in the tone test of FCT. **d** In the tone test of FCT, no significant difference in freezing time between the two groups at any time point. **e** Representative trajectories of each group in the Y-maze test. **f** Spontaneous alternation was significantly decreased in the Y-maze on days 1 and 3 following long-term isoflurane anesthesia. Exposure to the long-term isoflurane resulted in a marked increase in IL-1β (**g**) and IL-6 (**h**) levels in the hippocampus of mice (*n* = 5/group). Exposure to the long-term isoflurane resulted in a marked increase in IL-1β (**i**) and IL-6 (**j**) levels in astrocytes (*n* = 6/group). Data are presented as the mean ± SEM (*n* = 12 per group for behavioral assessments). Student’s *t*-test was used for statistical analysis. *p < 0.05; **p < 0.01; ***p < 0.001

In this study, we generated a cognitive dysfunction mouse model by inhaling isoflurane for 6 h and evaluated cognitive function at post-anesthesia days 1, 3, and 7 (Fig. 1a). The freezing time in the training phase of FCT was not significantly different between the two groups (Additional file 1: Fig. S2a; *t* = − 0.803, *p* = 0.427), indicating that the baseline level of learning and memory abilities were identical in different groups of mice. In the context test of FCT, long-term isoflurane anesthesia significantly decreased freezing time compared to the control condition in mice 1 and 3 days after isoflurane anesthesia (Fig. 2a and b; Day 1: *t* = − 2.837, *p* = 0.008; Day 3: *t* = 3.831, *p* = 0.001). On day 7, however, no more difference could be seen between the groups (Fig. 2a and b; *t* = 0.786, *p* = 0.437). In the tone test of FCT, no differences in freezing time were detected between groups at any time point (Fig. 2c and d; Day 1: *t* = 0.644, *p* = 0.524; Day 3: *t* = 0.276, *p* = 0.811; Day 7: *t* = 0.586, *p* = 0.562). The hippocampus-dependent memory was reflected by the context test, and the hippocampus-independent memory was reflected by the tone test. These results indicate impaired hippocampal functions in mice exposed to long-term isoflurane anesthesia.

To assess spatial working memory, mice were placed in a Y-maze and allowed to freely explore the three arms for 8 min. Movement velocity was not different between the two groups of mice, excluding defects in motor ability (Additional file 1: Fig. S2b; *t* = − 0.193, *p* = 0.848). All groups visited arms with the same rate (Additional file 1: Fig. S2c;* t* = − 0.150, *p* = 0.881). The percentage of spontaneous alternations was significantly lower among the Iso group compared to mice in the Con group on days 1 and 3 (Fig. 2e and f; Day 1: *t* = 2.093, *p* = 0.044; Day 3: *t* = 2.533, *p* = 0.016). No significant difference was found in the percentage of spontaneous alternations on day 7 between the Con and Iso groups (Fig. 2e and f; Day 7: *t* = 1.355, *p* = 0.184).

ELISA was applied to compare IL-1β and IL-6 expression in the hippocampus between Iso and control mice. Compared to control mice, IL-1β (Fig. 2g; Day 1: *t* = − 2.428, *p* = 0.030; Day 3: *t* = -2.264, *p* = 0.041; Day 7: *t* = 1.522, *p* = 0.152) and IL-6 (Fig. 2h; Day 1: *t* = − 2.366, *p* = 0.034; Day 3: *t* = − 2.990, *p* = 0.010; Day 7: *t* = 0.547, *p* = 0.594) expression in the hippocampus of mice in the Iso group was significantly increased on 1 and 3 days after anesthesia. Concomitantly, the level of IL-1β (Fig. 2i; *t* = − 2.613, *p* = 0.013) and IL-6 (Fig. 2j; *t* = − 3.840, *p* = 0.001) in isoflurane-treated primary astrocytes was significantly increased, suggesting activation of inflammatory response in isoflurane-treated astrocytes. These data suggest that long-term isoflurane anesthesia is capable of inducing neuroinflammation and cognitive deficits.

### Long-term isoflurane anesthesia inhibits GJs-Cx43 expression and impairs gap junctions function

**Fig. 3:**
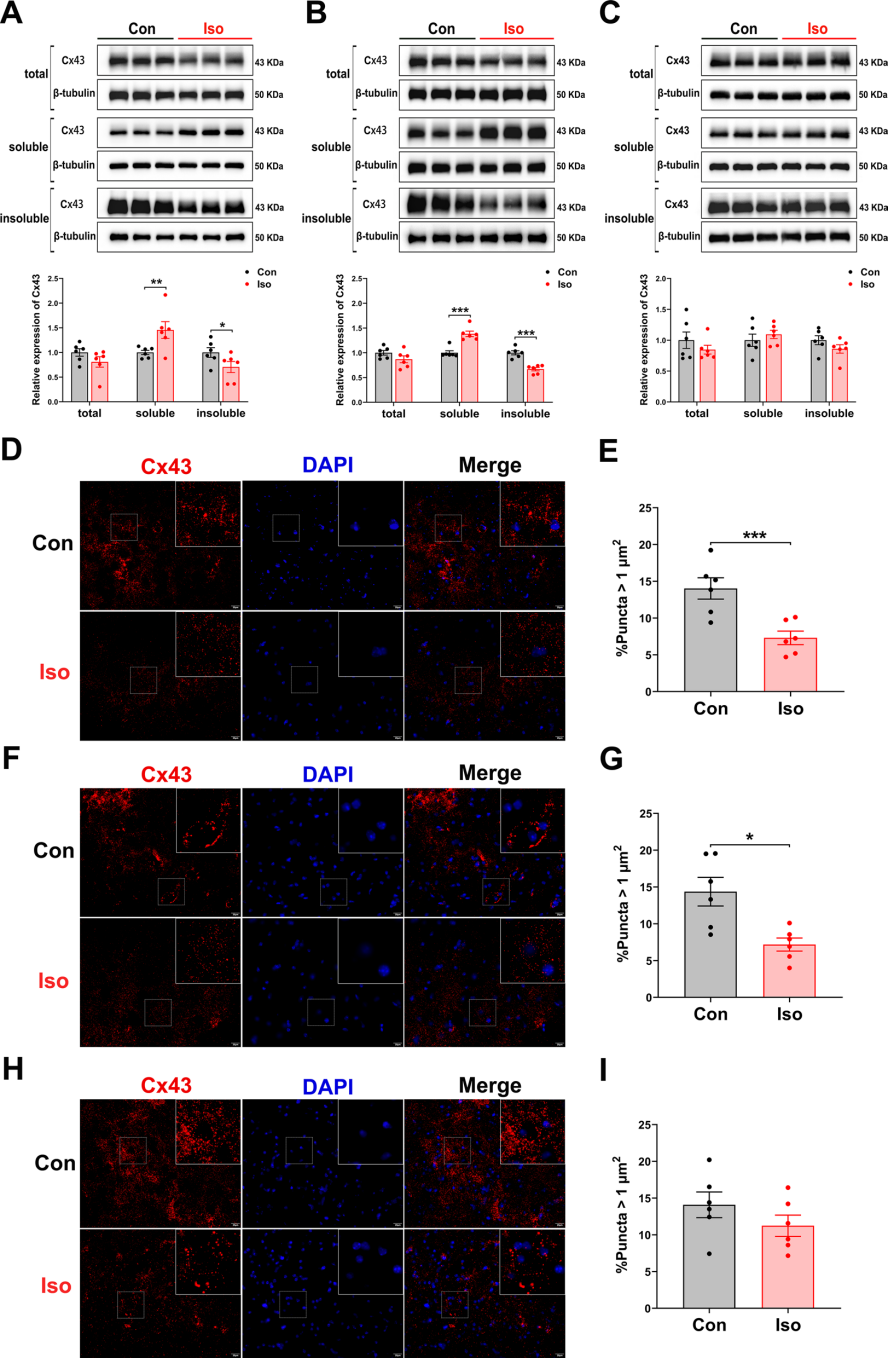
Cx43 protein expression patterns are altered in the hippocampus of mice exposed to long-term isoflurane anesthesia. **a** Representative protein bands shown for different configurations of Cx43 (upper panel) and their quantification (lower panel) are presented on day 1 post-anesthesia (*n* = 6/group). Representative bands of three mice per group were illustrated. **b** Representative bands shown for different configurations of Cx43 (upper panel) and their quantification (lower panel) are presented on day 3 post-anesthesia (*n* = 6/group). Representative bands of three mice per group were illustrated. **c** Representative bands shown for different configurations of Cx43 (upper panel) and their quantification (lower panel) are presented on day 7 post-anesthesia (*n* = 6/group), and three representative samples per group are shown. Representative fluorescent micrographs from the CA3 region of hippocampus (**d**) and quantification of GJs-Cx43 puncta (**e**) on day 1 post-anesthesia (*n* = 6/group). Representative fluorescent micrographs from the CA3 region of hippocampus (**f**) and quantification of GJs-Cx43 puncta (**g**) on day 3 post-anesthesia (*n* = 6/group). Representative fluorescent micrographs from the CA3 region of hippocampus (**h**) and quantification of GJs-Cx43 puncta (**i**) on day 7 post-anesthesia (*n* = 6/group). Data are presented as the mean ± SEM. Student’s *t*-test was used for statistical analysis. *p < 0.05; **p < 0.01; ***p < 0.001

We detected hippocampal Cx43 protein expression on days 1, 3, and 7 after anesthesia by Western blotting analysis. Compared with control mice at the same time points, long-term isoflurane exposure results in a reduction of total Cx43 level, yet this result was not statistically significant (Fig. 3a–c; Day 1: *t* = 1.850, *p* = 0.072; Day 3: *t* = 1.957, *p* = 0.057; Day 7: *t* = 1.119, *p* = 0.310). Given that Cx43 can form two functional conformations, gap junctions and hemichannels, we further examined the expression levels of Cx43 in these two different conformations. Differences also occurred concerning Cx43 configuration on days 1 and 3, GJs-Cx43 (insoluble fraction) expression levels were markedly decreased in the Iso group (Fig. 3a and b; Day 1: *t* = 2.639, *p* = 0.012; Day 3: *t* = 9.209, *p* < 0.001), while the levels of non-GJs-Cx43 (soluble fraction) were increased (Fig. 3a and b; Day 1: *t* = − 2.852, *p* = 0.007; Day 3: *t* = − 7.092, *p* < 0.001). On day 7, there were no significant differences in the expression of different conformations between the two groups (Fig. 3c; soluble fraction: *t* = − 1.280, *p* = 0.208; insoluble fraction: *t* = 2.015, *p* = 0.051).

To provide further evidence supporting the Cx43 conformational changes in hippocampal tissue of mice, we performed Cx43 immunofluorescence staining. Compared with control mice on days 1 and 3, the percentage of GJs-Cx43 fluorescent punctas was markedly diminished in Iso mice (Fig. 3d–g; Day 1: *t* = 6.567, *p* < 0.001; Day 3: *t* = 3.638, *p* = 0.013). The percentage of GJs-Cx43 fluorescent punctas was non-significantly reduced on day 7 (Fig. 3h and I; *t* = 1.891, *p* = 0.066). Altogether, the results described above suggested that the GJs-Cx43-mediated astrocytic networks are disrupted in the hippocampal tissue of mice with cognitive dysfunction.

**Fig. 4:**
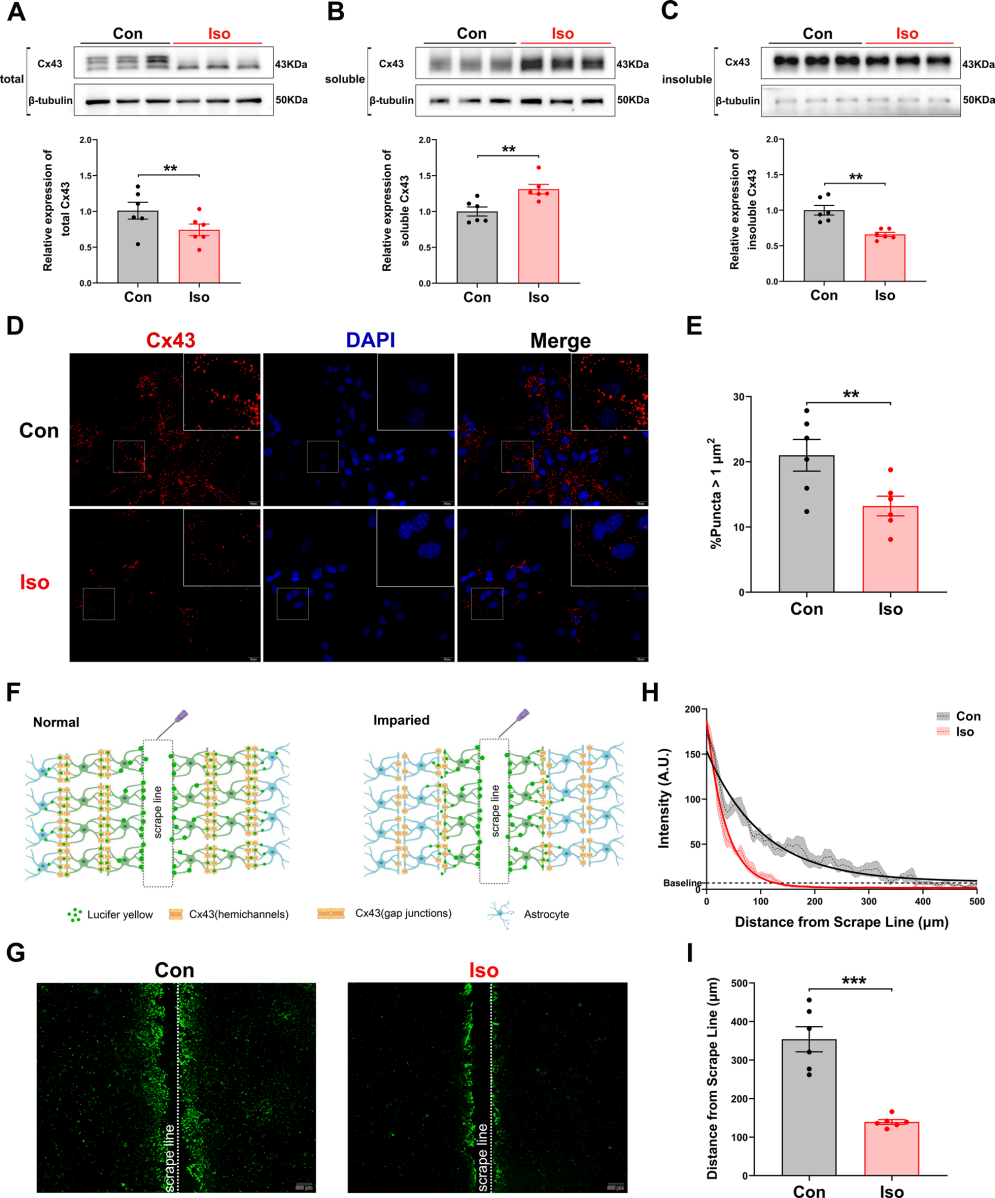
Long-term isoflurane anesthesia inhibits GJs-Cx43 expression, impairs gap junctions function, and causes inflammation in primary astrocytes. **a** A significant reduction of total Cx43 protein was observed in isoflurane-treated astrocytes. Upper panel showed representative immunoblot and lower panel were quantifications (*n* = 6/group). Three representative samples per group are shown. Cx43-soluble protein fractions was increased (**b**), while Cx43-insoluble protein fractions was decreased (**c**), in control astrocytes compared with isoflurane-treated astrocytes (*n* = 6/group). Three representative samples per group are shown. **d** Representative Fluorescent punctas represent GJs-Cx43 of control and isoflurane-treated astrocytes. Scale bar = 20 μm. **e** Quantification of the proportion of GJs-Cx43 punctas (*n* = 6/group). **f** The schematic diagram of scrape-loading dye transfer. The lucifer yellow can specifically stain cells through the GJs-Cx43, therefore, the longer the distance of lucifer yellow diffusion indicates the stronger the function of the gap junctions. **g** Representative lucifer yellow diffusion images in scrape-loading dye transfer. Scale bar = 200 μm. **h** The intensity of lucifer yellow and diffusion distance after long-term isoflurane exposure (*n* = 6/group). The dashed line represents the mean value of lucifer yellow fluorescence intensity at a specific distance, the shaded part represents SEM, and the solid line represents the fitted curve of lucifer yellow fluorescence intensity versus diffusion distance. **i **The yellow fluorescence diffusion distances of long-term isoflurane-treated astrocytes decreased noticeably (*n* = 6/group). Data are presented as the mean ± SEM. Student’s *t*-test was used for statistical analysis. *p < 0.05; **p < 0.01; ***p < 0.001

To further demonstrate the effect of long-term isoflurane exposure on gap junction-mediated astrocytic networks at the cellular level, a series of cell experiments were performed. Consistent with our in vivo findings, Western blot analysis confirms isoflurane-treated primary astrocytes had significantly reduced GJs-Cx43 expression (Fig. 4a–c; *t* = 3.025, *p* = 0.004; *t* = − 4.021, *p* = 0.001; *t* = 4.827, *p* = 0.003, respectively), and the immunofluorescence observation strengthened the Western blot results, which the percentage of GJs-Cx43 fluorescent punctas was markedly decreased in isoflurane-treated primary astrocytes (Fig. 4d and e; *t* = 3.511, *p* = 0.003).

Lucifer yellow can specifically diffuse through the gap junctions, thus the Lucifer yellow diffusion distance could reflect the functional state of gap junctions (Fig. 4f). Immediately after exposure to isoflurane for 6 h, we observed a significantly decreased lucifer yellow diffusion distance in primary astrocytes (Fig. 4g–i; *t* = 9.301, *p* < 0.001). These results suggest that long-term isoflurane exposure significantly impairs functional gap junction communication in mouse primary astrocyte cultures.

### ZP1609 can repair the astrocyte network by increasing Cx43 GJs conformation and attenuate the isoflurane-induced inflammatory response in primary astrocytes

To have a more definitive effect on gap junction-mediated astrocytic networks, we performed an intervention experiment with ZP1609 (specific enhancers of gap junction). First, to check the cytotoxicity of ZP1609, cell viability was measured in cultured primary astrocytes by a CCK-8 kit. After 30 min of treatment with ZP1609 at a concentration that was increased from 0 to 10 μg/ml, we verified that the cell viability in normoxia was not distinctly affected by various concentrations of ZP1609 (Additional file 1: Fig. S3a), in contrast, a significant improvement of cell viability of isoflurane-treated astrocytes was identified if 1 μg/ml ZP1609 were supplemented (Additional file 1: Fig. S3b). Previous studies also showed that 1 μg/ml was the optimal ZP1609 concentration for increasing the Cx43 gap junctions coupling [21]. Therefore, 1 μg/ml ZP1609 was applied for subsequent vitro experiments in this study.

**Fig. 5:**
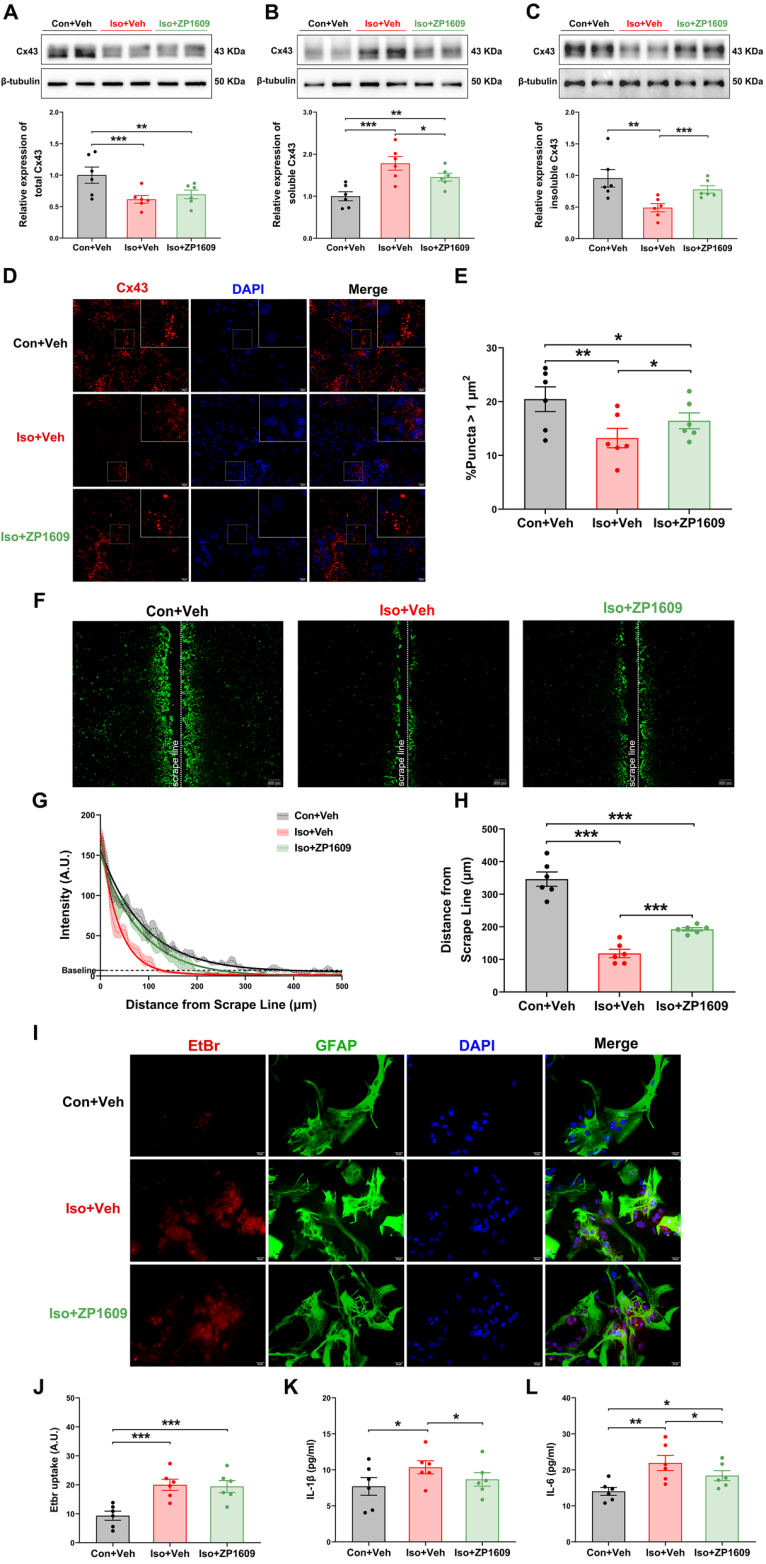
ZP1609 promotes GJs-CX43 expression and reconstitutes GJ-mediated astrocyte network, and inhibits the inflammatory response of primary astrocytes after receiving a long-term isoflurane anesthesia. **a** Upper panel showed representative total Cx43 protein bands in astrocytes for the different treatment groups and lower panel were quantifications (*n* = 6/group). Two representative samples per group are shown. **b** Upper panel showed representative Cx43-soluble protein fractions in astrocytes for the different treatment groups and lower panel were quantifications (*n* = 6/group). Two representative samples per group are shown. **c** Upper panel showed representative Cx43-insoluble protein fractions in astrocytes for the different treatment groups and lower panel were quantifications (*n* = 6/group). Two representative samples per group are shown. **d** Representative fluorescent punctas represent GJs-Cx43 of the different treatment groups. Scale bar = 20 μm. **e** Quantification of the immunofluorescence signals from the immunofluorescence microscopy experiments (*n* = 6/group). **f** Representative lucifer yellow diffusion images in scrape-loading dye transfer. Scale bar = 200 μm. **g** The intensity of lucifer yellow and diffusion distance. The dashed line represents the mean value of LH fluorescence intensity at a specific distance, the shaded part represents SEM, and the solid line represents the fitted curve of LH fluorescence intensity versus diffusion distance (*n* = 6/group). **h** Quantification of LH diffusion distance in different groups. **i** Representative images of EtBr uptake (red) of astrocyte (green) in different groups. **j** The relative fluorescence intensities of the astrocytes exposure to EtBr (*n* = 6/group). ZP1609 alleviated long-term isoflurane exposure-induced increased levels of IL-1β (**k**) and IL-6 (**l**) in primary astrocytes (*n* = 6/group). Data are presented as the mean ± SEM. ANOVA with Fisher’s PLSD test was used for statistical analysis. *p < 0.05; **p < 0.01; ***p < 0.001. Veh, vehicle (phosphate buffered saline)

Intriguingly, although ZP1609 increased the level of GJs-Cx43 expression, there was no significant difference in total Cx43 protein levels between the Iso + Veh group and Iso + ZP1609 group [Fig. 5a–c; *F*(2,15) = 8.818, *p* = 0.001; *F*(2,15) = 15.218, *p* < 0.001; *F*(2,15) = 14.764, *p* < 0.001, respectively]. Immunofluorescence staining further illustrated that the punctas representing GJs-Cx43 were increased in Iso + ZP1609 group compared with Iso + Vehicle group [Fig. 5d and e; *F*(2,15) = 6.520, *p* = 0.004]. As indicated by the dye transfer, the lucifer yellow diffusion distance in Iso + ZP1609 group was higher than Iso + Vehicle group, but it was lower than Con + Vehicle group [Fig. 5f–h; *F*(2,15) = 129.131, *p* < 0.001].

We next evaluated hemichannel activity in mouse primary astrocytes cultures after different treatments using EtBr uptake assay. A higher EtBr-positive signal indicates higher hemichannel activity. Although the control astrocytes showed only a faint intensity of EtBr signal, mouse primary astrocytes treated with isoflurane for 6 h presented significantly higher intensity of EtBr labeling (Fig. 5i and j; *F*(2,15) = 13.025, *p* < 0.001). Rather unexpected, there was no difference between Iso + Veh group and Iso + ZP1609 group in terms of the activity of hemichannel (Fig. 5j). In addition, ZP1609 also alleviated long-term isoflurane exposure-induced increased levels of IL-1β [Fig. 5k; *F*(2,15) = 3.588, *p* = 0.039] and IL-6 [Fig. 5i; *F*(2,15) = 8.902, *p* = 0.001] in primary astrocytes. These results suggest that long-term isoflurane exposure significantly impairs functional gap junctions communication while increasing hemichannels activity in mouse primary astrocyte cultures, and the gap junction uncoupling in astrocytes induced by long-term isoflurane exposure could be reversed by ZP1609.

### ZP1609 attenuates the isoflurane-induced hippocampal inflammatory response by repairing the astrocyte network via increasing Cx43 GJs conformation

To pinpoint the potential role of reconstructing Cx43 gap junction-coupled astrocyte networks on isoflurane-induced cognitive dysfunction, mice were therapeutically treated with ZP1609 under the long-term isoflurane anesthesia model (Fig. 1d). After establishing the model, mice were received three intraperitoneal ZP1609 injections (300 μg/kg) per day for three consecutive days and were killed after the behavioral assessments on day 3 post-anesthesia. The dose regimen was selected since it was previously reported in a C57BL/6 mouse model of ischemia/reperfusion [21], and in our pilot study, a single intraperitoneal administration ZP1609 (300 μg/kg) and multiple dosing regimen (1–2 days) did not improve performance on the tests of cognitive function in mice exposed to long-term isoflurane anesthesia (Additional file 1: Fig. S4).

**Fig. 6:**
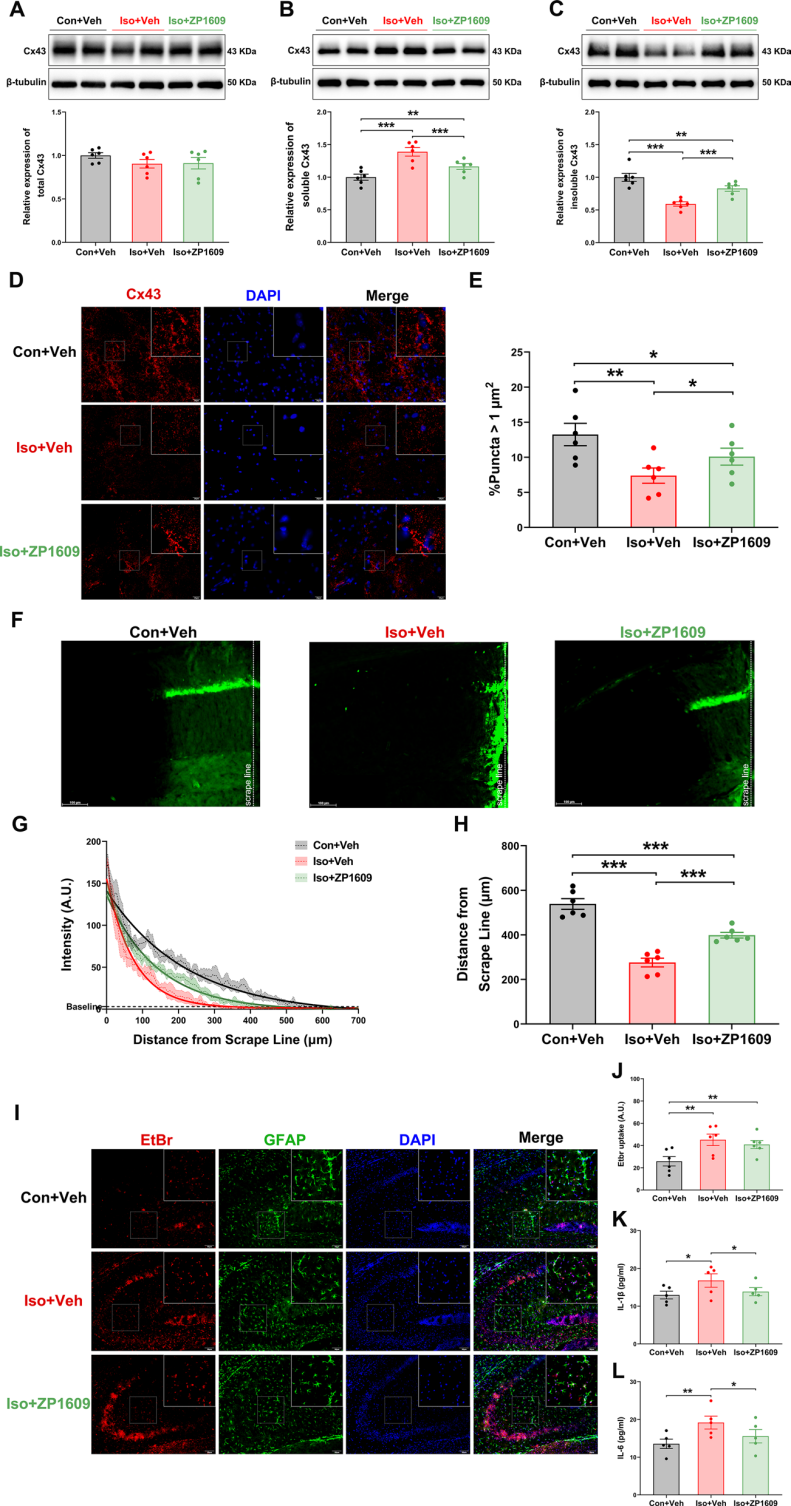
ZP1609 attenuates the isoflurane-induced hippocampal inflammatory response by repairing the astrocyte network via increasing Cx43 GJs conformation. **a** Representative protein bands of total CX43 in the hippocampus and its corresponding quantification (*n* = 6/group). Representative bands of two mice per group were illustrated. **b** Representative Western blots of soluble CX43 in the hippocampus and its corresponding quantification (*n* = 6/group). Representative bands of two mice per group were illustrated. **c** Representative Western blots of insoluble CX43 in the hippocampus and its corresponding quantification (*n* = 6/group). Representative bands of two mice per group were illustrated. **d** Representative immunofluorescence images for Cx43 in the CA3 region of hippocampus. **e** Quantification of the proportion of GJs-Cx43 punctas in hippocampus (*n* = 6/group). **f** Representative lucifer yellow dye transfer images from animals of all groups are illustrated. Scale bar = 100 μm. **g** The intensity of lucifer yellow and diffusion distance. The dashed line represents the mean value of lucifer yellow fluorescence intensity at a specific distance, the shaded part represents SEM, and the solid line represents the fitted curve of lucifer yellow fluorescence intensity versus diffusion distance (*n* = 6/group). **h** Quantification of lucifer yellow diffusion distance in different groups (*n* = 6/group). **i** Representative images of acute hippocampal brain slices (interaural = 1.86 mm, bregma = − 1.94 mm) showing astrocyte positive for GFAP (green) and EtBr uptake (red) in different groups. **j** The relative fluorescence intensities of the GFAP-positive cells in the CA3 area of hippocampus exposure to EtBr (*n* = 6/group). ZP1609 attenuates the elevation in IL-1β (**k**) and IL-6 (**l**) in the hippocampus resulting from long-term isoflurane anesthesia (*n* = 5/group). Data are presented as the mean ± SEM. ANOVA with Fisher’s PLSD test was used for statistical analysis. *p < 0.05; **p < 0.01; ***p < 0.001. Veh, vehicle (phosphate buffered saline)

Although ZP1609 significantly increased the proportion of GJs-Cx43 in the hippocampus, Cx43 absolute levels were not increased [Fig. 6a–c; *F*(2,15) = 1.208, *p* = 0.312; *F*(2,15) = 22.613, *p* < 0.001; *F*(2,15) = 37.197, *p* < 0.001, respectively], which was consistent with our in vitro results. Consistently, this finding was further supported by an immunofluorescence assay, the proportion of GJs-Cx43 in the hippocampus in Iso + Veh and Iso + ZP1609 group was lower than in Con + Veh group, while the proportion of GJs-Cx43 was higher in Iso + ZP1609 than in Iso + Veh group [Fig. 6d and e; *F*(2,15) = 8.387, *p* = 0.001].

The dye transfer assay revealed that the distance of diffusion in the hippocampus of Iso + Veh group mice was markedly reduced compared with Con + Veh group [Fig. 6f–h; *F*(2,15) = 86.716, *p* < 0.001]. Nonetheless, a notable increase of yellow fluorescent diffusion distance is observed upon ZP1609 treatment [Fig. 6f–h; *F*(2,15) = 86.716, *p* < 0.001], which indicates treatment with ZP1609 significantly reversed the effect of isoflurane exposure on intercellular coupling.

The activity of hemichannels was significantly increased in the Iso + Veh group and Iso + ZP1609 group compared with the Con + Veh group [Fig. 6i and j; *F*(2,15) = 7.688, *p* = 0.002]. Interestingly, consistent with our findings in vitro results, the activity of hemichannels did not differ between the Iso + Veh group and Iso + ZP1609 group (Fig. 6i and j). In addition, IL-1β [Fig. 6k; *F*(2,12) = 4.411, *p* = 0.022] and IL-6 [Fig. 6i; *F*(2,12) = 5.204, *p* = 0.012] expression in the hippocampus of mice exposed to long-term isoflurane anesthesia was significantly decreased by ZP1609.

### ZP1609 reversed ultrastructural abnormalities of astrocyte gap junctions in the hippocampus of Iso mice without affecting the tripartite synaptic structure

**Fig. 7:**
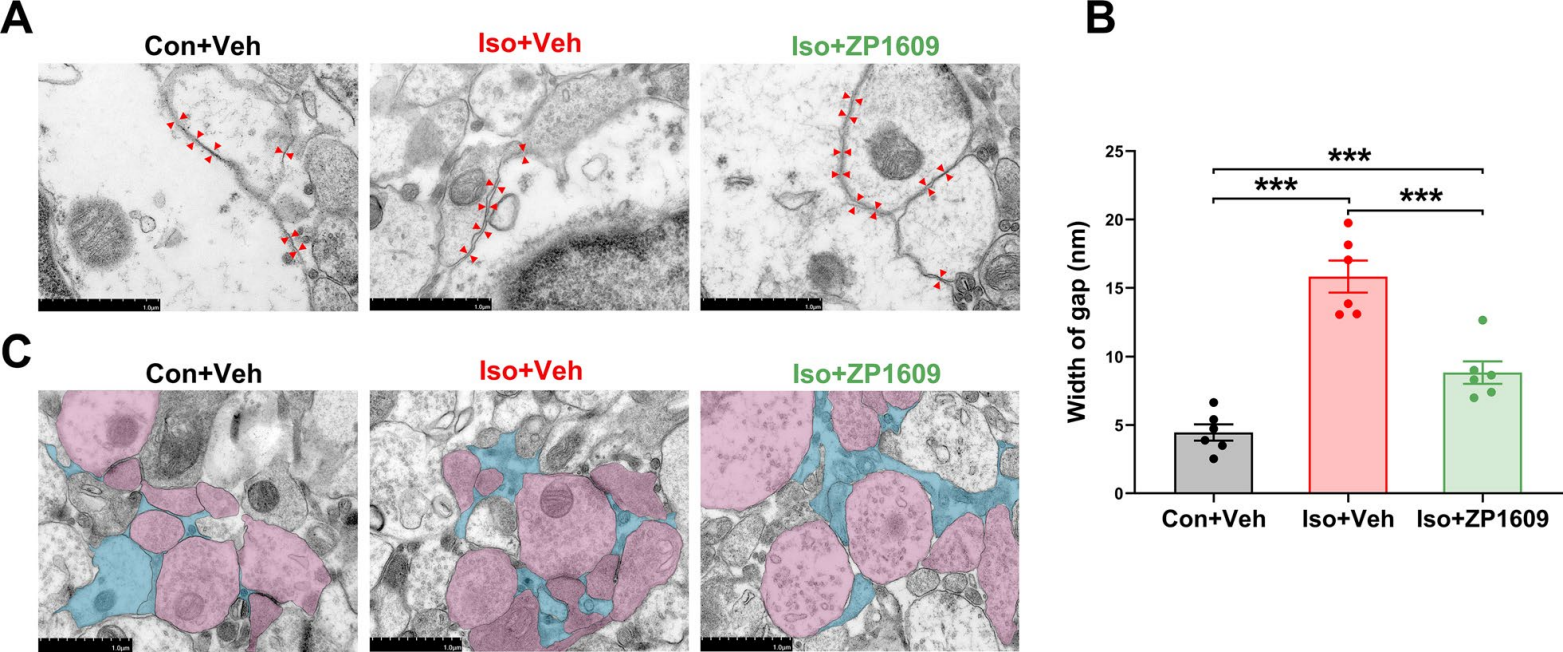
ZP1609 reversed ultrastructural alterations of astrocyte gap junction in the hippocampus of mice exposed to long-term isoflurane anesthesia without affecting the tripartite synaptic structure. **a** Electron micrographs showing astrocytic gap junction in the hippocampus of each group. Astrocytic gap junctions are indicated by red arrowheads. Scale bar = 1 μm. **b** The width of gap was significantly enlarged in mice exposed to long-term isoflurane anesthesia and this effect was reversed by ZP1609 treatment (*n* = 6/group). Data are presented as the mean ± SEM. ANOVA with Fisher’s PLSD test was used for statistical analysis. *p < 0.05; **p < 0.01; ***p < 0.001. **c** Astrocytes (cyan) make direct contact with neurons (lilac), forming tripartite synapses, where astrocytic processes are in close association with the presynapse and postsynapse at the synaptic junction. Scale bar = 1 μm. Veh, vehicle (phosphate buffered saline)

We used the width of the gap between two astrocytes as an index to quantify the ultrastructural alterations of the gap junctions, with a wider gap indicating more serious damage to the superstructure of gap junctions [37]. The significant broadened gap between two neighboring astrocytes was observed in Iso + Veh group and Iso + ZP1609 group compared with Con + Veh group, whereas the gap width was greatly reduced in mice treated with ZP1609 compared with Iso + Veh group [Fig. 7a and b; *F*(2,15) = 65.174, *p* < 0.001]. These results indicate that long-term isoflurane exposure impairs the structure of the astrocyte gap junction and ZP1609 has an amelioration effect.

The terminal processes of astrocytes envelop neuronal synaptic terminals, forming a tripartite synapse [38]. As displayed in Fig. 7c, the tripartite synapse involves three elements: presynaptic neurons, postsynaptic neurons, and perisynaptic astroglial processes (PAPs). This point-to-point form of communication between neurons and astrocytes based on tripartite synapses is a dynamic processor of the astrocyte network regulating neuronal activity. It is straightforward to see that tripartite synapse exists stably in different groups (Fig. 7c).

### The cognitive dysfunction of mice receiving long-term isoflurane exposure is mitigated by reconfiguring the Cx43 gap junction-coupled astrocyte network

We chose to perform behavioral assessments only on the third day after modeling because the mice were still in the treatment phase on the first day after modeling (Fig. 1d). Post-anesthesia day 7 was not investigated considering that no significant alterations occurred in GJs-Cx43 levels and cognitive function.

**Fig. 8:**
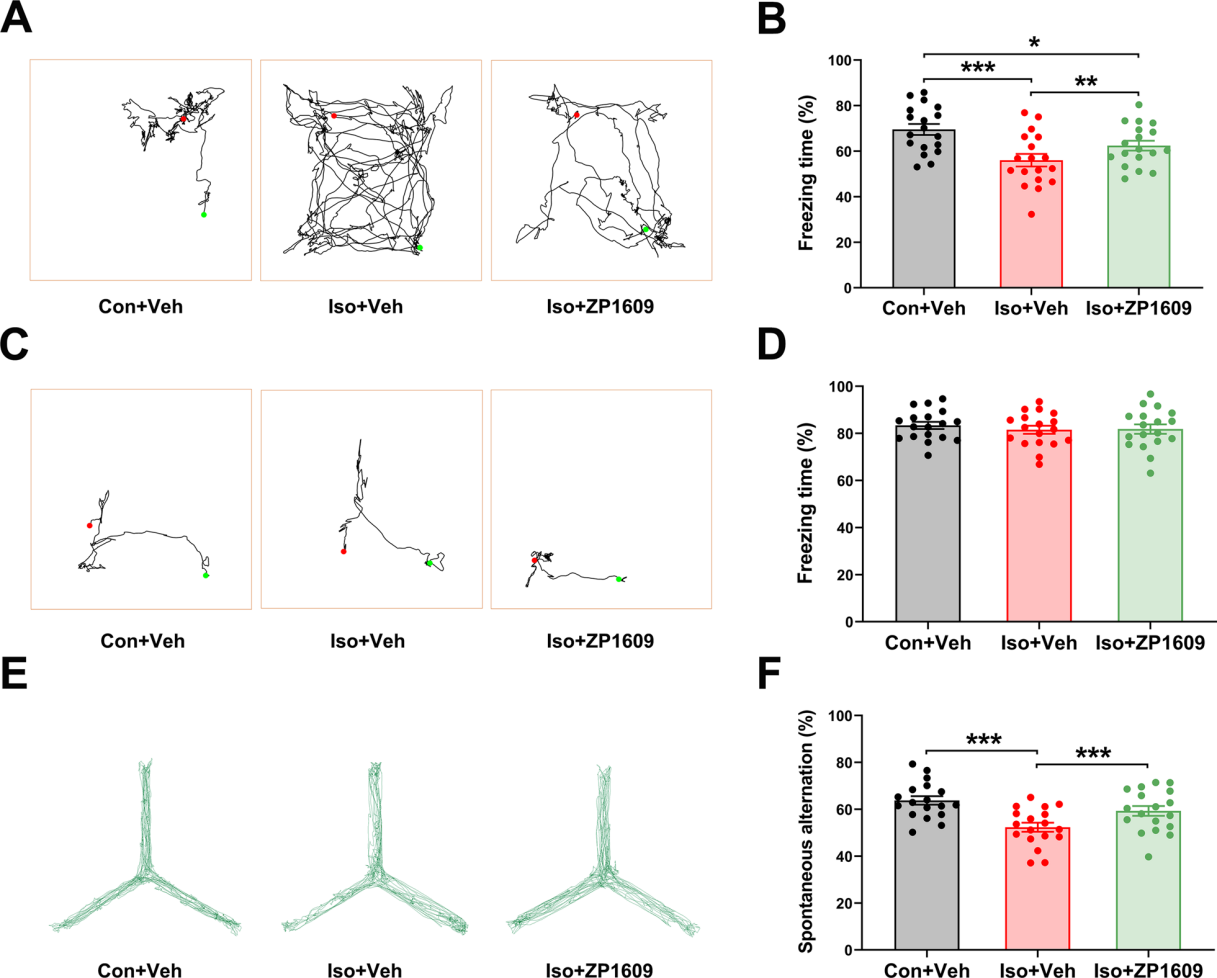
ZP1609 improves cognitive function in mice exposed to long-term isoflurane anesthesia. **a** Representative trajectories in different treatment groups in the context test of FCT. **b** Freezing time in the context test of FCT. A significantly higher freezing time was observed in the Iso + ZP1609 group, as compared to Iso and Iso + Vehicle group. **c** Representative trajectories in different treatment groups in the tone test of FCT. **d** Freezing time in the tone test of FCT and no differences in freezing time are observed among groups. **e** Representative trajectories of each group in Y-maze test. **f** The mice in the Iso + Vehicle group displayed lesser spontaneous alteration than the mice in the control group, which was reversed by the ZP1609 treatment. Data are presented as the mean ± SEM (*n* = 18 per group for behavioral assessments). ANOVA with Fisher’s PLSD test was used for statistical analysis. *p < 0.05; **p < 0.01; ***p < 0.001. Veh, vehicle (phosphate buffered saline)

In the training phase of FCT, the baseline level of learning and memory abilities were identical in different groups of mice [Additional file 1: Fig. S5a; *F*(2,51) = 0.519, *p* = 0.597]. In the context test of FCT, the freezing time was significantly decreased in the Iso + ZP1609 group and Iso + Veh group compared to the Con + Veh group, while administration of ZP1609 significantly increased the freezing time in Iso mice [Fig. 8a and b; *F*(2,51) = 11.292, *p* < 0.001]. In the tone test of FCT, no differences in freezing time were detected among groups [Fig. 8c and d; *F*(2,51) = 0.382, *p* = 0.684]. In the Y-maze test, there was no significant difference in movement velocity [Additional file 1: Fig. S5b; *F*(2,51) = 1.556, *p* = 0.216] and visited arms rate [Additional file 1: Fig. S5c; *F*(2,51) = 0.177, *p* = 0.838] among different groups. The mice in the Iso + Veh group were displayed lesser spontaneous alteration than the mice in Con + Veh group, which was reversed by the ZP1609 treatment [Fig. 8e and f; *F*(2,51) = 13.154, *p* < 0.001]. The above-described results suggest that the gap junction-mediated astrocytic networks are damaged in the hippocampus of Iso mice, however, reconstructing the astrocytic networks by increasing the gap junction conformation of Cx43 reduces hippocampal neuroinflammation and improves cognitive function in mice exposed to long-term isoflurane anesthesia.

## Discussion

**Fig. 9:**
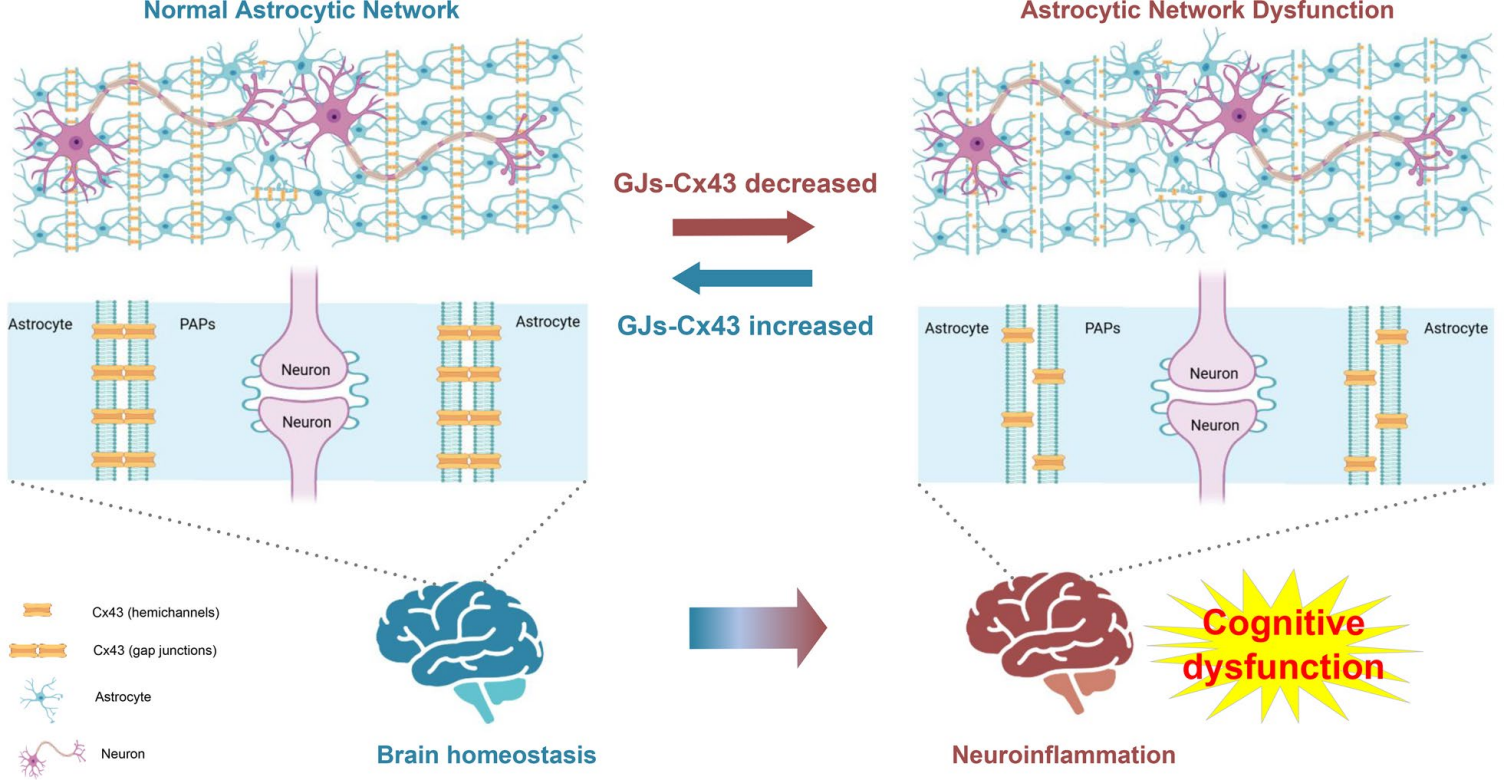
General overview of the main highlights of this study. Cx43 is the most predominant expression connexin isoform in astrocytes. Cx43 forms both gap junctions (GJs) and hemichannels at the astrocyte surface. Gap junctions form, when two Cx43 in the membrane of neighboring cells align. Astrocytes are well connected through GJs-Cx43, forming an astrocytic network that bears an essential role in maintaining metabolic homeostasis in the brain. Long-term isoflurane anesthesia induced a down-regulation of GJs-Cx43, which subsequently impaired astrocytic networks, leads to an amplification of the insult signal (e.g., neuroinflammation) and brain homeostatic imbalance, and eventually contributes to the development/progression of POCD. Remodeling of gap junction-mediated astrocytic networks by treatment with ZP1609 significantly reduced central neuroinflammation and improved cognitive function in mice exposed to long-term isoflurane anesthesia. PAPs: perisynaptic astrocytic processes

In the present study, by combining molecular biology, cytology, animal ethology, and electron microscopy, we identified Cx43 in astrocytes as a key molecular determinant of astrocytic network efficacy and hippocampus-dependent contextual memory. Long-term isoflurane anesthesia decreased GJs-Cx43 expression in the hippocampus, and the reduced GJs-Cx43 levels could further contribute to defects in astrocytic networks, trigger or exacerbate neuroinflammation and eventually lead to cognitive impairments in mice. Of note, the GJs-Cx43 enhancer ZP1609 resulted in gap junction-mediated astrocytic network reconstruction, a reduction in neuroinflammation, and improved cognitive function. This indicates that the Cx43 gap junction-mediated astrocytic network is likely involved in the pathogenesis of isoflurane-induced cognitive dysfunction (Fig. 9).

Postoperative cognitive dysfunction (POCD) is an advancing field of research in anesthesiology and neuroscience and was first reported in 1955 [39]. Since then, a large number of studies have been carried out to explore the molecular mechanisms underlying the pathogenesis of POCD and to establish new diagnostic methods and therapeutic interventions. Previously, all forms of postoperative cognitive impairments were called POCD; however, there have been increasing calls to develop specific terminology that could be used for cognitive classifications in the general population when investigating cognitive changes after anesthesia and surgery. In November 2018, the Perioperative Cognition Nomenclature Working Group recommended that “Perioperative Neurocognitive Disorders (PND)” should be used as an overarching term for cognitive impairment identified during the preoperative or postoperative periods [10]. This change aligns PND with the phenotypically similar neurocognitive diagnoses listed in the Diagnostic and Statistical Manual of Mental Disorders, version, such as Alzheimer’s disease [10]. PND includes cognitive decline diagnosed before the operation, postoperative delirium, delayed neurocognitive recovery, and POCD. In this study, we just focused on the cognitive changes during the perioperative period, more specifically, cognitive decline induced by long-term isoflurane anesthesia. Given its complex pathophysiology and heterogeneous etiology, the development of POCD could involve a complex interplay of patient‐related factors, environmental factors, and iatrogenic factors. Neuroinflammation is a prominent feature and a likely contributor to POCD pathogenesis [40]. It has been reported that the occurrence of cognitive disturbances upon CNS inflammation has been correlated with increased levels of IL-6 and IL-1β [41–43]. In the present study, we showed that the hippocampal levels of the pro-inflammatory cytokines IL-1β and IL-6 were all increased by exposure to isoflurane, which was consistent with previous studies [44, 45], and suggesting that the hippocampus is susceptible to neuroinflammation induced by long-term isoflurane anesthesia.

Astrocytes are the most abundant cell type in the human brain and account for one-third of the cells in the murine CNS [46]. Astrocytes are actively involved in normal memory functions as well as in abnormal processes leading to cognitive impairment in pathological conditions [47–50]. Simultaneously, as the major contributor to neuroinflammation, astrocytes can release different neuroactive substances (inflammatory cytokines and chemokines) that recruit immune cells into the CNS to promote and maintain inflammatory responses [46]. In contrast, astrocytic control of cognitive function goes awry in neuroinflammatory conditions. For example, the local increase of TNF-α in the hippocampal dentate gyrus activates astrocyte TNF receptor type 1, which in turn triggers persistent alterations of excitatory synapses, contributing to memory deficits in a mouse model of multiple sclerosis [42]. Astrocyte-mediated neuroinflammation has been demonstrated to play an important role in the progression of POCD [14]. Notably, previous studies have demonstrated that isoflurane significantly inhibits astrocyte activity [51] and ion channel function [52] as well as alters the ability of cultured astrocytes to support neuronal growth [53]. Our study also confirmed that could inhibit the activity of astrocytes, and found that long-term isoflurane exposure resulted in higher concentrations of IL-1β and IL-6 in primary astrocytes. These results suggest that targeting astrocyte pathways may represent an important new therapeutic opportunity to fight against cognitive alterations or decline in many CNS diseases.

Cx43 has two functional forms: gap junctions and hemichannels. Cx43 hemichannels have been shown to release toxic molecules into the extracellular space [54] and induce neuroinflammation [55, 56]. Under normal conditions, Cx43 hemichannels are thought to be closed or inactive, whereas pathologic insult can cause pathologic hemichannel opening [57, 58]. Therefore, Cx43 hemichannels are often seen as “pathological pores”, while GJs are seen as “physiological pores”. Given the existence of two functional forms of Cx43, the change in total Cx43 does not fully reflect the functional status. For example, decreased, unchanged and increased Cx43 expression have been reported in epileptic tissue [59]. Therefore, in our study, we examined the Cx43 conformation in these two different functional states separately and found that long-term isoflurane anesthesia caused both a decrease in GJs-Cx43 and an increase in Cx43 hemichannel activity. Notably, a previous study suggested that inhibition of the Cx43 hemichannel can alleviate internal fixation of tibial fracture-induced cognitive impairment in aged mice [60]. This is not inconsistent with our findings because the two conformational changes of Cx43 (the decrease in GJs and increase in hemichannels) indicate a disruption of the astrocyte network [61]. These results confirmed that there is a decrease in intercellular gap junctional communication with a concomitant increase in hemichannel activity in the pathological processes of POCD.

ZP1609, also known as danegaptide or GAP-134, was originally shown to function in myocardial protection and antiarrhythmia by promoting Cx43 gap junctions in cardiomyocytes [62, 63]. In parallel with the intense study of cardioprotective mechanisms, current studies have demonstrated that ZP1609 can cross the blood–brain barrier (BBB) in preclinical models of ischemic–reperfusion injury and has an important role in the maintenance of normal brain function and protection against pathological insults [21, 64]. BBB disruption also has been uniformly reported in several rodents models of POCD [65, 66], Further, isoflurane anesthesia can disrupt the integrity of the BBB, resulting in increased permeability [67, 68]. Notably, ZP1609 injected intraperitoneally has the ability to cross the intact BBB [69]. Therefore, in our study, intraperitoneal administration of ZP1609 could enter the brain through the BBB and function as anticipated. An interesting phenomenon in this study is that the ZP1609 promoted Cx43 to form gap junctions but did not reduce the enhancement of Cx43 hemichannel activity induced by long-term isoflurane anesthesia, and a similar phenomenon was also observed by Freitas-Andrade et al. [21] Indeed, the non-GJs-Cx43 (soluble fraction) comprising cytoplasmic Cx43, Cx43 connexons in the plasma, and Cx43 en route to degradation [27, 35]. Therefore, the increases in the gap junctions functionality and the decrease in the non-GJs-Cx43 indicate that the main mechanism of ZP1609 should involve a change in the functionality of connexin proteins already present in the cells and/or promote the formation of GJs without affecting the Cx43 hemichannel activity. Therefore, agents that simply target Cx43 might not have the expected therapeutic effect for POCD, and it might be more meaningful to explore agents that can specifically block hemichannels or promote the maintenance of gap junctions. On the other hand, ZP1609 is applicable for relieving postoperative cognitive dysfunction in patients with arrhythmia seems to be reasonable from the perspective of potential clinical translation.

Gap junctions-coupled astrocytic networks could be a double-edged sword. From a metabolic perspective, the redistribution of resources through the gap junctional network seems to be neuroprotective. From a buffering perspective, the network allows astrocytes to regulate the extracellular environment more effectively. However, because of the nonselective nature of these gap junctions, there is also the potential that they mediate the spread of damaging cues [70]. One plausible explanation is that GJ-mediated networks may be both protective and deleterious. GJs can protect dying astrocytes to some extent by transferring ions and essential metabolites from healthy astrocytes to dying neighbors [71]. In contrast, dying astrocytes can also transfer harmful ions and metabolites to neighboring healthy ones via the remaining gap junctions, which can again cause damage to their neighbors [72]. Noteworthy, GJs close under extreme conditions such as high intracellular calcium ion concentration, low intracellular pH, and strong trans junctional voltage differences, to prevent death signals from being transferred through the astrocytic network [73–75]. This strongly suggests that the function of GJs changes with pathological progression. There is a delicate balance in the function of GJs. Depending on the balance, there can be two different outcomes: bystander damage and saving neighbors. Extrapolation of our findings to other disease models, given the dual effects of GJs, should be done with caution. New studies are necessary to elucidate the specific roles of CJs in different periods of pathological processes and to define the correct therapeutic window according to the specific stage of the pathology.

The importance of astrocytes in the CNS is further exemplified, as they are closely related to neurons, forming tripartite synapse, which actively regulates the excitability and plasticity of neurons [76, 77]. Peripheral astrocyte processes (PAPs) reach lengths comparable to some neurites, and one astrocyte territory can contact up to 100,000 synapses [78]. Astrocytic perisynaptic processes cover approximately 50–60% of all synapses in the CNS, with particularly high coverage of complex excitatory synapses [79, 80]. These structural features emphasize the importance of the astrocytic network for maintaining homeostasis and normal function of the brain. Notably, Semyanov et al. argue that the expanding knowledge on physiological interactions of cellular and noncellular components of nervous tissue necessitates advancing the concept of a “tripartite synapse” to a concept named the “active milieu” [81]. The “active milieu” unifies all components of nervous tissue (e.g., neuronal and glial compartments, extracellular space, extracellular matrix, and vasculature) into a dynamic information processing system. However, as the most numerous neuroglial cells in the CNS, astrocytes are also the primary concern of this conceptual framework. Astrocytic PAPs are dynamic structures that affect the degree of synaptic coverage by changing their morphology, thus influencing synaptic transmission. Lifelong analysis demonstrates that astrocytes undergo biphasic morphological changes—the astrocytic domain and complexity increase from juvenile to middle age, with subsequent decreases in old age [82]. Furthermore, it has been established that astrocyte coupling through gap junctions and the volume fraction of perisynaptic processes (PAPs) decrease significantly with age [77]. This might explain, in part, why elderly patients have higher rates of POCD. Although we found that the structural features of tripartite synapses are still present in mice exposed to long-term isoflurane anesthesia, their functional variations remain to be explored in depth. Therefore, follow-up studies are needed to study the impact of tripartite synapse on POCD by employing a more direct analysis of synaptic microenvironments.

The limitations of this study deserve consideration. First, none of these studies involved aged animals, given the vulnerability of aged mice as was the limited numbers of animals. However, advanced age is an acknowledged risk factor for POCD development. Future studies must be conducted in aged mice in order to confirm the results obtained in our study. Second, although there were no differences in cognitive function and Cx43 conformation in anesthetized mice compared to control mice on the 7th day after long-term isoflurane anesthesia, the changes of GJs and hemichannels activity still warranted further investigation.

## Conclusions

In conclusion, we provide compelling evidence that disruption of GJs-Cx43-mediated astrocytic network is associated with isoflurane-induced cognitive dysfunction and reveal that GJs-Cx43-mediated astrocytic network remodeling can restrain neuroinflammation and improve cognitive function. Neuroinflammation is a major hallmark of progressive neurodegenerative disorders, including POCD. Our study shed new light on the pathophysiology of POCD, and GJs-Cx43 may therefore constitute a novel target for therapeutic intervention in neurodegenerative diseases.

## Supplementary Information

**Additional file 1: Figure S1.** Primary astrocytes were identified by GFAP immunostaining. **Figure S2.** Behavioral assessment by Fear condition test (FCT) and Y-maze after long term isoflurane anesthesia. **Figure S3.** The effect of ZP1609 at different concentrations on the astrocyte viabilities. **Figure S4.** Behavioral effects of different dosing schedules of ZP1609 on PND mice. **Figure S5.** Behavioral assessment by Fear condition test (FCT) and Y-maze after ZP1609 treatment.

## Data Availability

The data supporting the findings of this study are presented within the manuscript.

## Abbreviations

BBB: Blood–brain barrier
Cx43: Connexin 43
CNS: Central nervous system
FCT: Fear conditioning test
GJs: Gap junctions
IL-1β: Interleukin 1-beta
Iso: Isoflurane
POCD: Postoperative cognitive dysfunction
PND: Perioperative neurocognitive disorders
PAPs: Perisynaptic astrocytic processes
TNF-α: Tumor necrosis factor-alpha
Veh: Vehicle
