# Supplementary material for: Connexin 43 gap junction-mediated astrocytic network reconstruction attenuates isoflurane-induced cognitive dysfunction in mice
Source: J Neuroinflammation. 2022 Mar 7;19:64. doi: 10.1186/s12974-022-02424-y (PMC8903726; doi:10.1186/s12974-022-02424-y)
Supplement: Supplementary file 1 — Additional file 1: Figure S1. Primary astrocytes were identified by GFAP immunostaining. Figure S2. Behavioral assessment by Fear condition test (FCT) and Y-maze after long term isoflurane anesthesia. Figure S3. The effect of ZP1609 at different concentrations on the astrocyte viabilities. Figure S4. Behavioral effects of different dosing schedules of ZP1609 on PND mice. Figure S5. Behavioral assessment by Fear condition test (FCT) and Y-maze after ZP1609 treatment. [file 12974_2022_2424_MOESM1_ESM.pdf]

Additional file

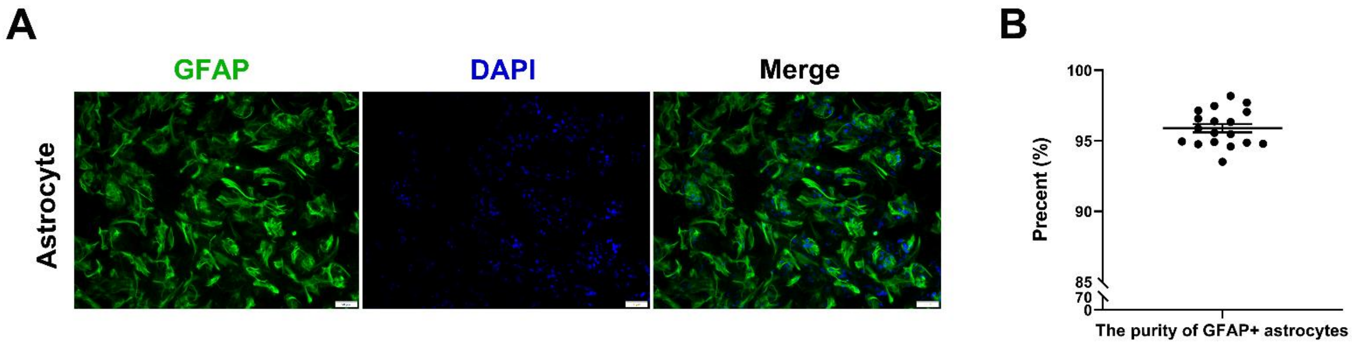

Fig. S1 Primary astrocytes were identified by GFAP immunostaining. (**a**) Representative images showing GFAP-positive astrocytes. The cell nuclei were counterstained with DAPI. (**b**) Merge files showed a purity of astrocytes was 95.90 ± 0.30% (mean ± SEM). Each data point was obtained from different cultures established from 18 breeding pairs.

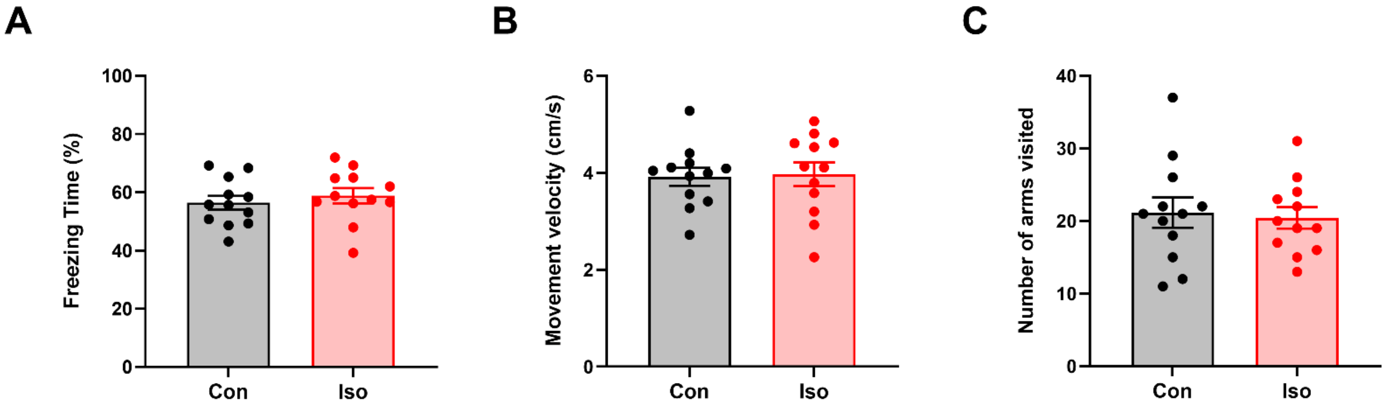

Fig. S2 Behavioral assessment by Fear condition test (FCT) and Y-maze after long term isoflurane anesthesia. (**a**) The freezing time among different group mice in the training phase of FCT. (**b**) The average locomotion velocity of mice in the Y-maze. (**c**) The number of arms visited in the Y-maze session. Data are presented as the mean ± SEM (n = 12). Student's t-test was used for statistical analysis.

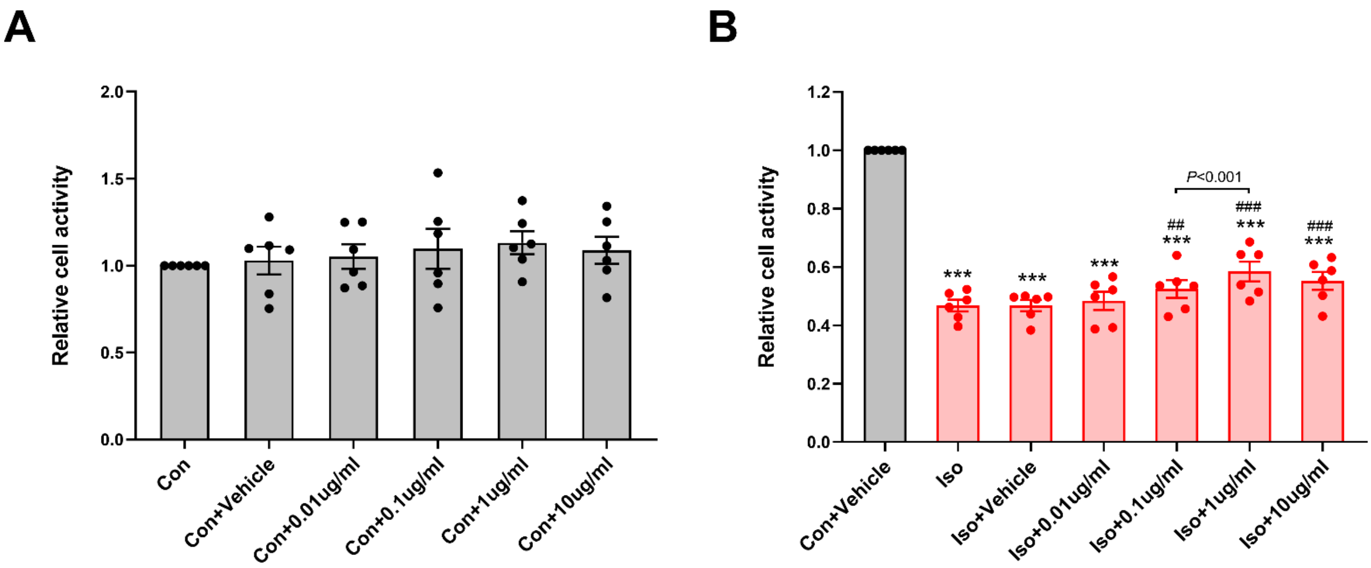

Fig. S3 The effect of ZP1609 at different concentrations on the astrocyte viabilities. (**a**) The cell viability in normoxia was not distinctly affected by various concentrations of ZP1609. (**b**) The cell viability of isoflurane-treated astrocytes improved significantly when cells were treated with 1μg/ml ZP160. Data are presented as the mean ± SEM (n=6/group). ANOVA with Fisher's PLSD test was used for statistical analysis, $^{*}P<0.05$, $^{**}P<0.01$ compared with Con+Vehicle; $^{\#}P<0.01$, $^{\#\#}P<0.01$ compared with Iso+Vehicle. Vehicle = phosphate buffered saline.

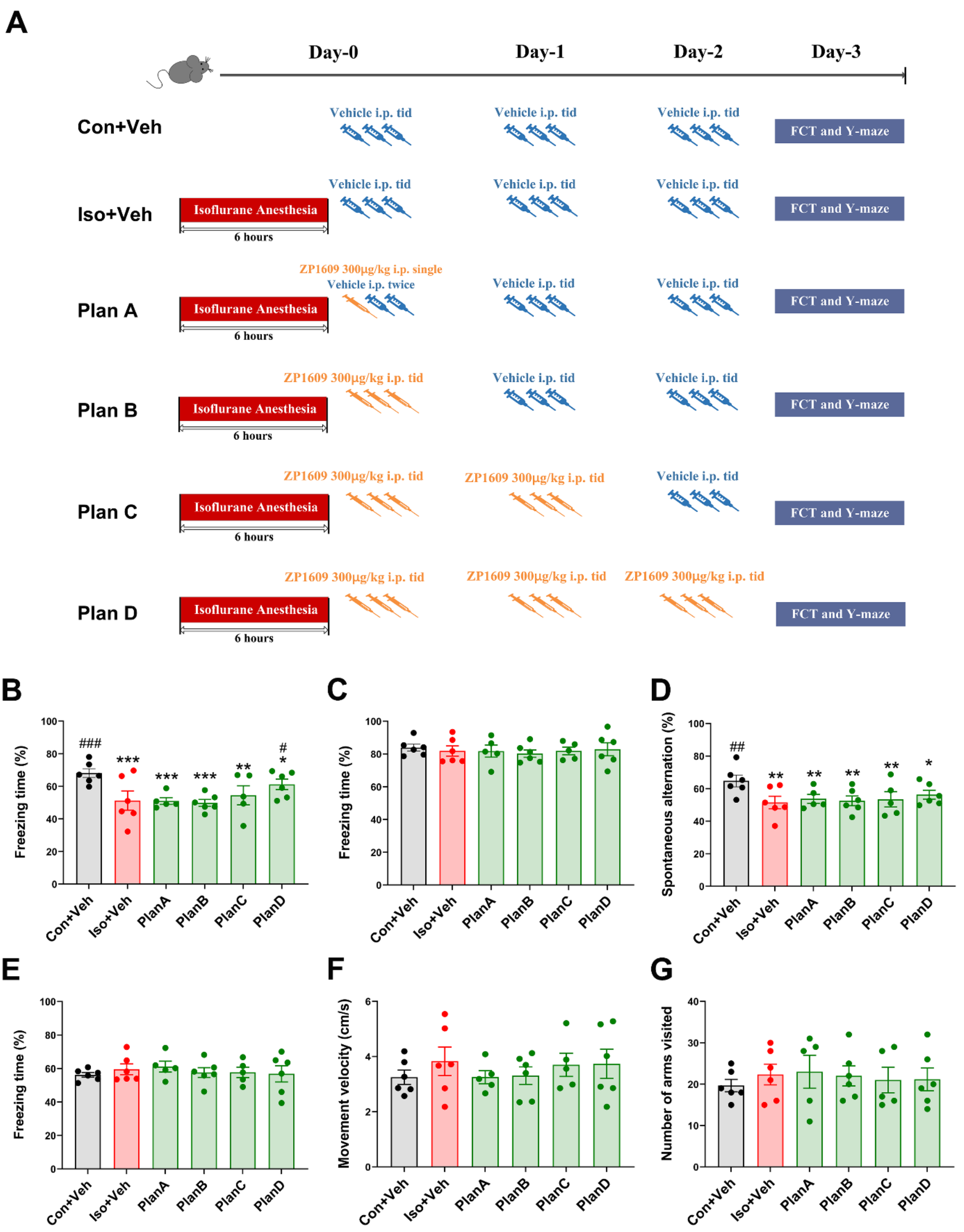

Fig. S4 Behavioral effects of different dosing schedules of ZP1609 on PND mice. (**a**) Schedule of ZP1609 administration and flow chart of the trial. (**b**) The freezing time of the groups 3 days after anesthesia in the context test of FCT. The freezing time was clearly increased in the Plan D mouse group compared to the Iso+Veh group. (**c**) In the tone test of FCT, no significant difference in freezing time among different groups. (**d**) Spontaneous alternation ratio in Y maze test. (**e**) The freezing time among different group mice in the training phase of FCT. (**f**) The average locomotion velocity of mice

in the Y-maze. (**g**) The number of arms visited in the Y-maze session. Data are presented as the mean ± SEM (n = 6 for Con+Veh, Iso+Veh, Plan B, and Plan D; n=5 for Plan A and Plan C). ANOVA with Fisher's PLSD test was used for statistical analysis, **P*<0.05, ***P*<0.01, ***P*<0.001 compared with Con+Veh; $^{\#}P$<0.01, $^{\#\#}P$ <0.01, $^{\#\#\#}P$ <0.01 compared with Iso+Veh. Veh, Vehicle (phosphate buffered saline).

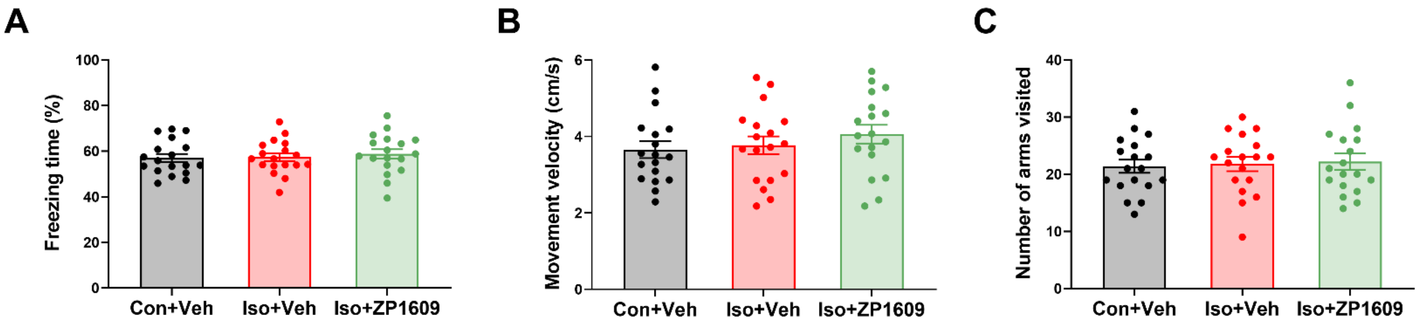

Fig. S5 Behavioral assessment by Fear condition test (FCT) and Y-maze after ZP1609 treatment. (**a**) The freezing time among different group mice in the training phase of FCT (n = 18/group). (**b**) The average locomotion velocity of mice in the Y-maze (n = 18/group). (**c**) The number of arms visited in the Y-maze session. Data are presented as the mean ± SEM (n = 18/group). ANOVA with Fisher's PLSD test was used for statistical analysis. Veh, Vehicle (phosphate buffered saline).
